# Statistically principled feature selection for single cell transcriptomics

**DOI:** 10.1186/s12859-025-06240-y

**Published:** 2025-10-02

**Authors:** Emmanuel P. Dollinger, Kai Silkwood, Scott Atwood, Qing Nie, Arthur D. Lander

**Affiliations:** 1 Center for Complex Biological Systems, University of California, Irvine, Irvine, CA 92697 USA; 2 Department of Developmental and Cell Biology, University of California, Irvine, Irvine, CA 92697 USA; 3 Department of Mathematics, University of California, Irvine, Irvine, CA 92697 USA

**Keywords:** Fano factor, Clustering, Cell type, Cell state, Rare cell identification, Single cell transcriptomics, Feature selection

## Abstract

**Background:**

The high dimensionality of data in single cell transcriptomics (scRNAseq) requires investigators to choose subsets of genes (“feature selection”) for downstream analysis (e.g., unsupervised cell clustering). The evaluation of different approaches to feature selection is hampered by the fact that, as we show here, the difficulty of feature selection can vary greatly, depending on the dataset being analyzed.

**Results:**

For routine cell type identification, even randomly chosen features can perform well, but for cell type differences that are subtle, both number of features and selection strategy matter strongly. We present a simple feature selection method grounded in an analytical model that allows for interpretable delineation of how many and which features to choose, facilitating identification of biologically meaningful rare cell types. We compare this method to default methods in scanpy and Seurat, as well as SCTransform, showing how greater accuracy can often be achieved with surprisingly few, well-chosen features.

**Conclusions:**

Feature selection is a critical step in scRNAseq for downstream analyses. We explore the pitfalls that can arise from incautious feature selection and present a statistical method to facilitate improved outcomes.

**Supplementary Information:**

The online version contains supplementary material available at 10.1186/s12859-025-06240-y.

## Background

Single cell RNA sequencing (scRNAseq) measures the transcriptional profiles of individual cells, enabling cell type classification, lineage inference, and elucidation of experimental differences in both gene expression and cell type abundance [1–7]. As cell type identity is generally not known *a priori*, unsupervised clustering is commonly used to group transcriptomically similar cells. As a first step, genes (“features”) considered most likely to be markers of cell type or state are selected and used for subsequent dimensionality reduction and clustering [8].

In general, limiting features in high dimensional data to those that are most informative for downstream applications improves interpretability, increases computational efficiency, prevents overfitting, and improves the performance of clustering algorithms [9]. However, in scRNAseq, there is no accepted definition of “most informative.” Most widely used algorithms calculate gene expression variation across cells, but they differ significantly in how variation is measured, as well as how the appropriate number of features is determined [10–19]. Although some comparisons have been made of the effects of different feature selection methods on clustering [13, 20, 21], it occurred to us that performance of a selection method will likely depend on aspects of the dataset to which it is applied. These could include how many cell types are present, their relative abundance, and the number and magnitude of gene expression differences between cells of different types. To our knowledge, the interaction of these factors with different methods for ranking and selecting genes has not been explored in a systematic way.

Here we show that, for datasets in which the desired goal is to cluster relatively abundant cells that differ greatly in gene expression, how features are selected is almost irrelevant. Even random sets of genes, if large enough, tend to perform nearly as well as algorithmically chosen features. In more demanding situations, such as when clustering cells with similar gene expression, we identify cases in which both the method of feature selection and the number of features selected markedly influence clustering outcomes.

This led us to revisit the question of how best to identify genes for which cell-to-cell variation is greater than otherwise expected. Starting with an analytical model for the distribution of un-normalized scRNAseq data, we developed a method to quantify the magnitude of biologically meaningful gene expression variation and to estimate the probability that such variation occurs by chance. This method, which we call BigSur (*B*asic *I*nformatics and *G*ene *S*tatistics from *U*nnormalized *R*eads), provides a theoretical framework for scRNAseq data analysis which enables both feature selection and the inference of gene regulatory networks from gene-gene correlations (the latter use having been outlined elsewhere [22]). Here we show that using BigSur for feature selection enables identification of biologically relevant groups of cells while minimizing loss of discriminatory power due to the use of excessive numbers of features.

## Results

### For common tasks, random sets of genes can perform nearly as well as algorithmically-chosen features.

Existing feature selection algorithms often choose up to several thousand genes for use in cell clustering (e.g [19, 23–25]). As this can represent a substantial fraction of the expressed genome, one is left to wonder how many of these features are necessary, and whether the number chosen is particularly important.

**Fig. 1:**
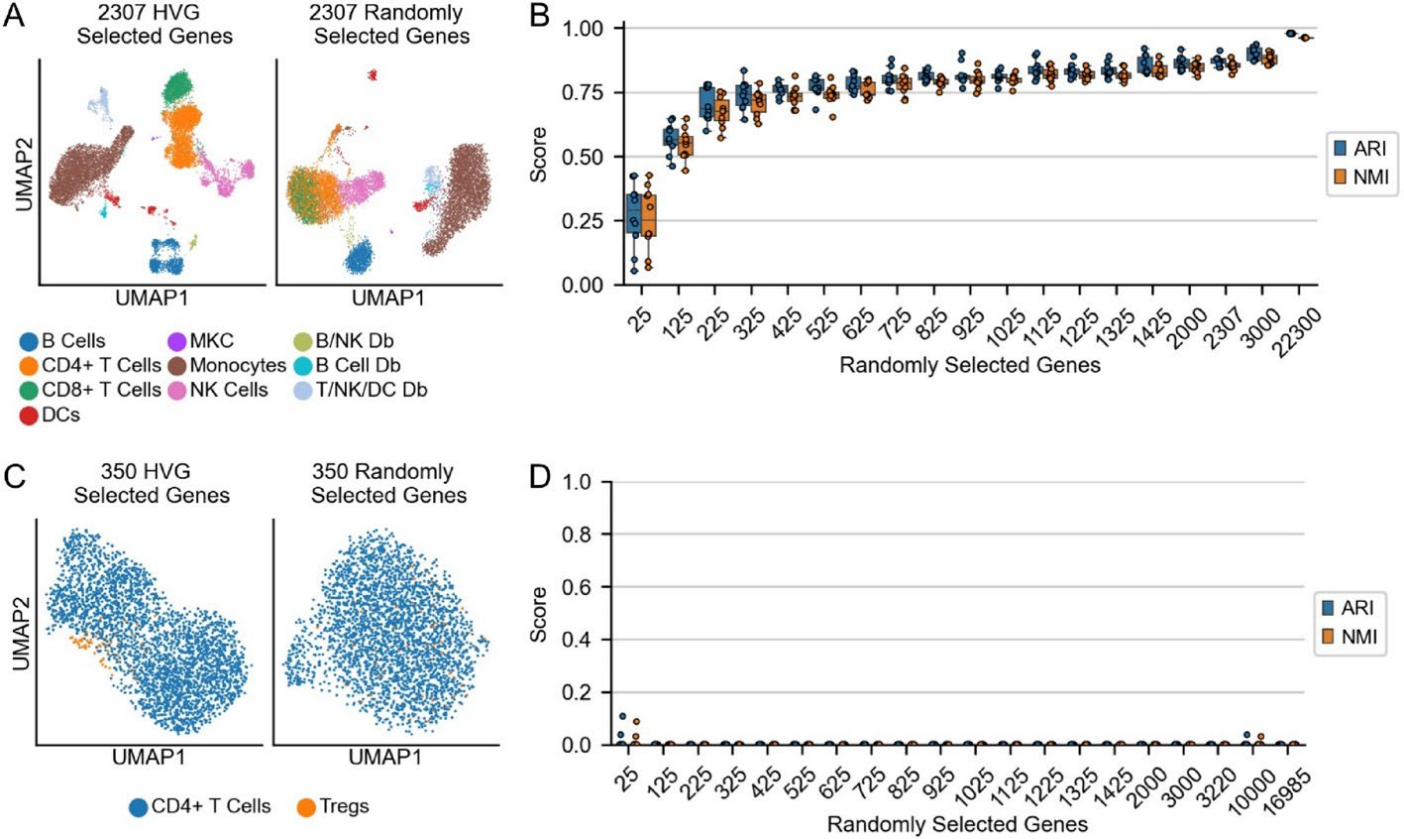
Performance of randomly selected genes as features. **A** UMAPs of PBMCs calculated using either HVG-selected genes (left) or the same number of randomly selected genes (right). **B** Adjusted Rand index (ARI) and normalized mutual information (NMI) of prediction of cell type as a function of number of genes selected. The predictions were generated by a linear Support Vector Machine (SVM), trained on the top principal components (PCs) calculated on randomly selected genes. Ten sets of random genes were selected at each step. **C** UMAP of CD+ T cells, including the T regulatory cell (Tregs) subset, calculated using either HVG-selected genes (left) or same number of randomly selected genes (right). **D** ARI and NMI of Treg identification, as a function of number of randomly selected genes. The predictions were generated by a linear Support Vector Machine (SVM) as in panel B. Twenty sets of random genes were selected at each step.

In common scenarios, in which cell types of interest are relatively abundant and well separated in gene expression space, we find that features chosen at random often perform nearly as well as those selected by popular algorithms. We illustrate this by clustering the 10k cell PBMC (peripheral blood mononuclear cell) dataset from 10x Genomics, which is commonly used for methods evaluation. Initially, we used scanpy’s default feature selection method (highly variable genes, “HVGs”), with cutoffs and parameters set to their default values. HVGs bins genes by normalized mean expression, calculates a z-score for each gene’s Fano factor (variance / mean) relative to the other genes’ Fano factors in the same bin, and ranks genes by those z-scores (“normalized dispersion”) [18]. Using the 2,307 genes chosen by HVGs, cells were clustered according to the default pipeline: calculating the first 50 principal components (PCs) using principal component analysis (PCA), creating a nearest neighbor graph in PCA space, and assigning clusters on that graph using the Leiden algorithm. Clusters were then labeled based on the expression of known marker genes. As expected, cell groups were well separated on a UMAP plot (Fig. 1A, left; see also Fig. S1).

For comparison, we used an equal number of genes (2,307) chosen at random as features and performed the same task. As shown in Fig. 1A, right, the same cell types grouped well and separated similarly from each other, with the one exception that some of the CD4+ and CD8+ T cells were intermixed.

We next asked how many random genes were needed to cluster cells correctly, and how repeatable the results were. Since, in this case, labels are already assigned to each cell, we can treat this question as a supervised problem. We therefore set as ground truth the cell types shown in Fig. 1A and assessed the ability of a straightforward classifier (a linear support vector machine, or SVM) to identify the cell types from the 50 top PCs of randomly selected genes (see methods). We first trained a classifier on 2,307 randomly selected genes (the number of genes selected by default by HVGs) and found that, with the exception of one of the doublet clusters, the classifier correctly identified at least 70% of each cell type (Fig. S2A).

We then asked how the SVMs would perform with varying numbers of selected genes. We first varied the number of genes selected by HVGs, retraining the SVM at each step, and concluded that, as long as at least 725 HVGs-selected genes are used, the adjusted Rand index (ARI) and the normalized mutual information (NMI)–two common clustering metrics–will be above 0.95 (Fig. S2B). We then performed the same task, varying the number of randomly selected genes from 25 to 22,300 (all genes present in the dataset), testing 10 samplings at each number and retraining the SVM at each step. As the numbers of randomly selected genes increased, both the ARI and NMI increased quickly to an ARI of 0.8 at 725 random genes and an NMI of 0.8 at 925 random genes (Fig. 1B). Both scores continued to rise up to a maximum of 0.98 and 0.96 for ARI and NMI, respectively, when using all genes as features. Across the 10 trials, the variance in the ARI and NMI scores decreased as the number of genes increased but was already relatively low by 525 random genes (Fig. 1B).

To verify that the number of cells belonging to each cell type didn’t substantially influence these results, we also downsampled the dataset to only contain 1,000 cells of each cell type and again trained SVMs on increasing numbers of randomly selected genes. We again found that ARI and NMI scores of the predictions increased to 0.77 and 0.79, respectively, with 725 randomly selected genes, and continued to rise up to 0.97 and 0.96 with all genes (Fig. S2C, D).

These results suggest that almost any random set of genes of size greater than a few hundred is potentially sufficient to correctly classify groups of cells that are well separated in gene expression space. Although we are not aware of this observation having been explicitly noted before, it supports the view that the effective dimensionality of gene expression is far lower than the number of expressed genes [26–28]. In other words, any random sample of reasonable size would be expected to include a substantial number of genes that correlate with all major patterns of gene expression.

### Some tasks are sensitive to choice of feature selection algorithm

Given the results above, the task displayed in Fig. 1A is clearly not suitable for assessing the performance of any feature selection algorithm, as the lack of an algorithm (using all genes) and a trivial algorithm (random gene selection) perform reasonably well.

This led us to consider more challenging tasks, for example one involving more subtle gene expression differences. We therefore subsetted the PBMC dataset to just the CD4+ T cells and performed unsupervised clustering. Using 350 features selected by HVGs, we could identify a FOXP3+ T regulatory cell (Treg) cluster of approximately 1.8% (53/2,908) of the cells (Fig. 1C, left; Treg identification is shown in Fig. S2E, F). Tregs are a well described CD4+ T cell subtype, regulating immune responses in many biological systems [29]. In this case, using an equal number of random genes as features, the Treg population was not identifiable (Fig. 1C, right). There was a small population of cells, visible in the lower left quadrant of the UMAP calculated using randomly selected genes, that separated from the main group, but this separation seemed to be driven by the substantially lower sequencing depth of those cells, rather than significant differences in gene expression (Fig. S2G).

Even using much larger numbers of genes—up to all 16,985 expressed genes—and testing 20 random gene sets for each sample size, ARI and NMI scores remained close to zero (Fig. 1D). Thus, in a sufficiently difficult task, HVGs performed much better than random.

### Number of features can influence success or failure in unpredictable ways

The fact that random gene selection failed to identify Treg cells, even when the entire expressed transcriptome was used, emphasizes that a good feature selection method must not only include enough genes that are predictors of cell types or states, but also exclude enough genes that are not. Indeed, even with features ranked by HVGs, the use of too many features substantially decreased calculated ARI and NMI scores (Fig. S2H). Such behavior reflects a well-known phenomenon in machine learning, in which the accuracy of a classifier initially rises with increasing number of features and then falls [30]. This happens because, in high dimensions, inclusion of features that have low predictive power can degrade the effects of data with high predictive power (i.e., the “noise” can overwhelm the “signal”) [31].

Because of this, it is important that feature selection algorithms for scRNAseq not only order genes by their utility as features, but also decide how far down the list one should go, i.e., the number of features to use when performing unsupervised clustering. Currently, popular algorithms typically choose feature number based either on arbitrary variability cutoffs or on calculations that depend upon arbitrarily adjustable hyperparameters.

To assess how important these choices are, we selected three clustering tasks: subclustering CD4+ T cells (as in Fig. 1C, D); CD8+ T cells (also from the PBMC 10k dataset), and human retinal amacrine cells [32]. We selected a particular cluster of interest in each dataset, shown in Fig. S3A–C, with the aim of determining how the number of features used influences the Leiden clustering algorithm’s ability to isolate the cell state of interest. We therefore varied the number of features by starting from those considered by HVGs to be most highly variable and adding an increasing number of features from further down the list. To assess clustering performance, we defined a *purity score*: the fraction of target cells within the cluster containing the most target cells (see methods).

**Fig. 2:**
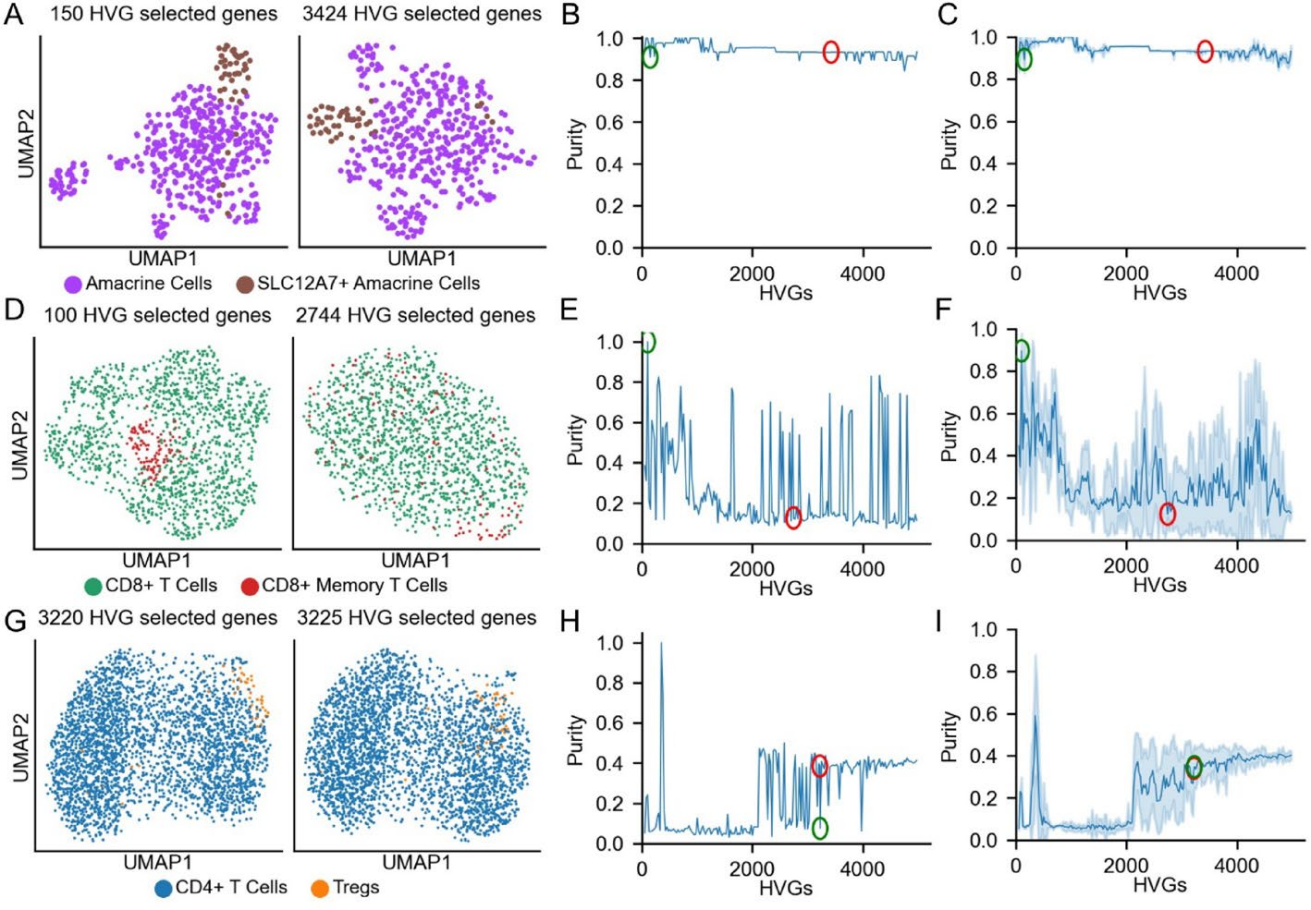
Numbers of features and feature selection method influence clustering. **A** UMAPs of amacrine cells and SLC12A7-expressing amacrine cells using either 150 HVGs (left) or the default number of HVGs (3,424; right). **B** Purity score (see main text) for SLC12A7+ cells using different numbers of features selected by HVGs. Green and red circles show results using 150 and 3,424 genes, respectively. **C** Mean and standard deviation of purity scores for SLC12A7+ cells, using 50 randomly selected starting seeds for the Leiden clustering algorithm. **D** UMAPs of CD8+ T cells and memory CD8+ T cells using 100 HVGs (left) and the default number of HVGs (right). **E** Purity score of memory CD8+ T cells with increasing number of HVGs. Green and red circles mark enrichment scores at 100 HVGs and the default number of HVGs (2,744), respectively. **F** Mean and standard deviation of purity scores of memory CD8+ T cells using 50 different Leiden starting seeds, as in C. **G** UMAPs of CD4+ T cells and regulatory T cells (Tregs) using either 3,220 genes (left) or default number of HVGs (right, 3,225). **H** Purity score of Tregs as in panels B and E. **I** Mean and standard deviation purity scores of 50 randomly selected Leiden starting seeds, as in panels C and F.

As shown in Fig. 2, the outcomes of clustering differed markedly among the three tasks. With amacrine cells, a population of *SLC12A7*-expressing cells could be cleanly identified with either 150 or 3,424 features, the latter representing HVG’s default selection (Fig. 2A). In this case, purity was relatively insensitive to the number of features used (Fig. 2B). Since the Leiden clustering algorithm requires a starting seed, for each set of features, we used 50 randomly chosen starting seeds, and calculated purity scores for each case (Fig. 2C) [33].

With CD8+ T cells, we identified a subpopulation of memory T cells (a CCL5+ population equaling 9.3% of the total) using the top 100 HVG features (Fig. 2D). Here, performance degraded markedly when larger numbers of features were used and sometimes declined to very low levels (Fig. 2E). Indeed, when the default number of features suggested by HVGs was used (2,744; red circle in Fig. 2E), the ability to identify memory T cells as a distinct cluster was lost. We found that the observed “choppiness” in panel 2E—where purity scores jump from high to low with the addition or subtraction of just a few features—reflected a high sensitivity in this particular dataset to the choice of random starting seed used by the Leiden clustering algorithm (Fig. 2F).

Finally, we turned again to the CD4+ T cells. The Treg population previously discussed in Fig. 1C mostly grouped together when the default number of 3,220 HVGs was used (Fig. 2G, H and H red circle), but adding in even 5 more features destroyed the ability to identify this cluster (Fig. 2H, green circle). We also noted that the purity score of the CD4 dataset, when using the default starting seed of the Leiden algorithm, was markedly higher at 350 genes (the “spike” visible in panel H) than the average purity score with the same number of genes. This suggests that the cells in the Treg cluster identified in Fig. 1 were clustered together at least partially due to a lucky choice of starting seed.

As one might expect, variability in clustering success due to changes in the number of selected features has a substantial effect on common downstream tasks, such as differential expression analysis. To illustrate this, we first clustered the CD8+ T cells using 100 HVGs and determined the differentially expressed genes (DEGs) using the Benjamini-Hochberg FDR-corrected *p*-values of the Mann-Whitney-U test. We then repeated this task using the default number of HVGs (2,744) and compared the outcomes. Changing the number of HVGs changed both the number of clusters identified and the genes identified as DEGs (Fig. S4). In particular, the expression of *CCL5*, which marked CD8+ memory T cells when using 100 HVGs, no longer specifically marked any cluster when using the default number of HVGs. In addition, the magnitudes of differential expression tended to be larger in the clusters assigned using 100 HVGs than in those assigned using the default number of genes.

Taken together, the results in Fig. 2 indicate that, for tasks for which having an algorithm for feature selection matters, selecting the right number of features can affect whether cells are correctly clustered together. Too many features can be as harmful as too few, and for some datasets, even small changes in feature number can have dramatic consequences. Importantly, feature numbers chosen by commonly used procedures under default conditions could lead to systematic misclassification. We therefore asked whether there might be a principled way to optimize the joint tasks of finding features and determining how many to use.

### A statistical approach to feature selection

Common feature selection methods tend to choose genes by the degree to which they are variable within a dataset, with differences among methods typically involving the way data are initially transformed, the way variability is defined and measured, and how the appropriate number of features is determined [19, 23, 24]. Ideally, one would want to select features that are more variable than expected by chance, but this requires knowledge of the expected gene expression distribution when all cells are biologically equivalent (i.e., the only sources of variability being technical and biological noise), which we refer to as the “null” distribution. Because there has long been a lack of general agreement on the appropriate null distribution for scRNAseq data, it has become common to empirically determine an appropriate model from data, sometimes with additional modifications to produce a better fit (e.g., SCTransform) [17–19].

We chose the alternative approach of constructing a null distribution analytically, based on assumptions about how scRNAseq data arise. Specifically, we assume that biological noise—random fluctuations in transcript numbers in identical cells—follows a log-normal distribution, which agrees with both theoretical predictions and empirical observations in mammalian cells [34, 35]. In addition, we assume that the technical noise associated with cell preparation, library preparation, sequencing, etc., can simply be approximated as sampling noise, i.e., by the Poisson distribution. This agrees with a number of recent observations and arguments in the scRNAseq literature [50, 51].

We thus write the following model:

$$\:{x}_{ij}\sim Poisson \left(\textit{Log-Normal}\left(\mu_{j},c_{j}\right)\right)$$

where ${\mu}_{j}$ is the expected number of transcripts of gene $\:j$, $\:{c}_{j}\:$is the coefficient of variation of that gene, and ${x}_{ij}$ is the observed counts (UMIs) detected for gene $j$ in cell $\:i$. Note that we parametrize the log-normal distribution in terms of its actual mean and coefficient of variation, and not the mean and coefficient of variation of the underlying normal distribution from which it may be derived.    

**Fig. 3:**
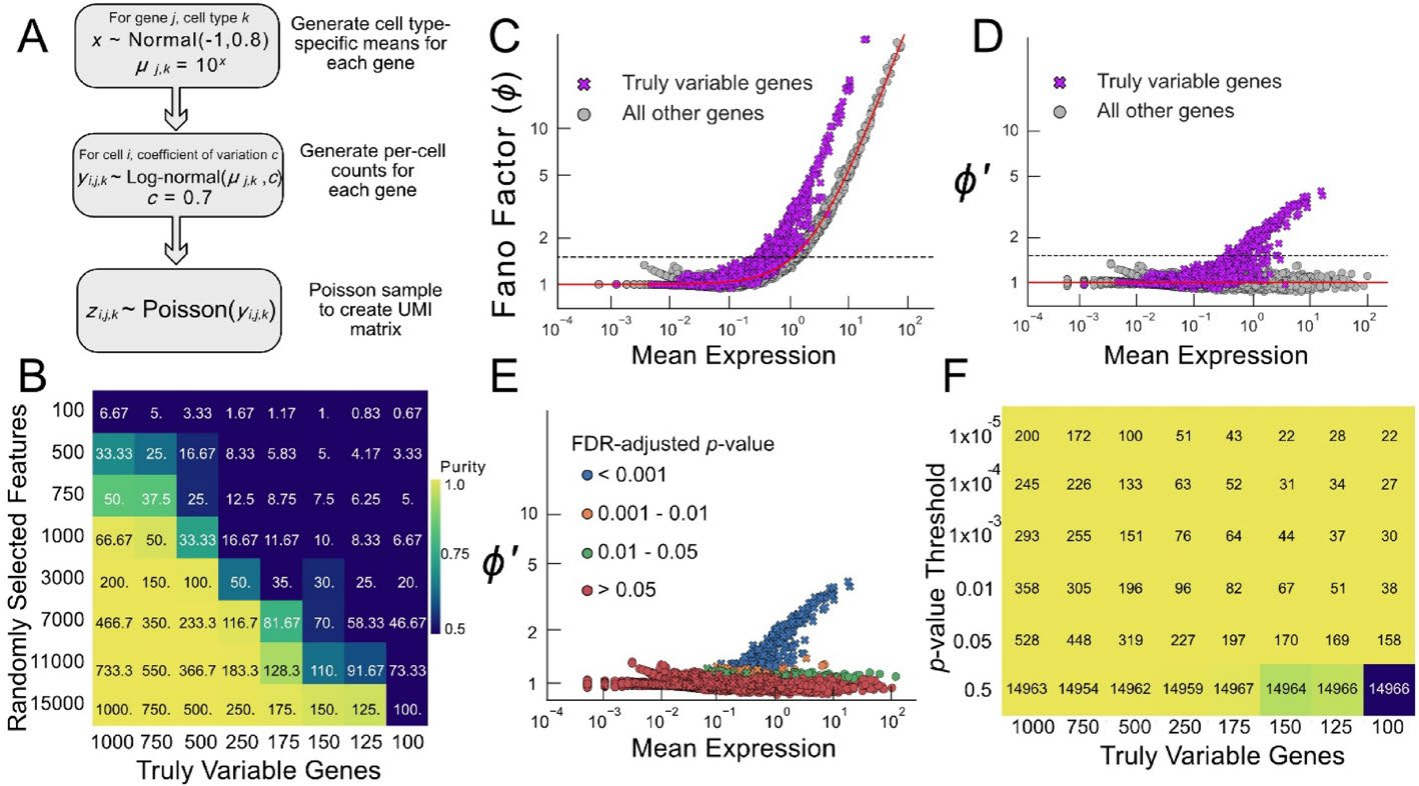
BigSur identifies truly variable features in simulated data. **A** Simulated data generation pipeline (see methods). **B** Average purity score of simulated datasets, clustered using varying numbers of randomly selected genes. Each purity score is the average of ten rounds of random feature selection. The numbers on each tile indicate the expected number of truly variable genes to be found among the selected features (the proportion of genes that were selected of the total number of genes multiplied by the number of truly variable genes). **C** Fano factor ($\varphi$ ) of simulated data with 1,000 truly variable genes plotted against their means. Orange crosses indicate genes that were truly variable. Blue points are genes that were selected from the same distributions for the two cell types. The red line depicts the expected relationship, under the null hypothesis, between the Fano factor and mean expression. The dashed line at $\varphi=1.5$ illustrates the large number of non-truly-variable genes that would be chosen if the Fano factor is used to select features. **D** Observed relationship between the modified Fano factor ($\varphi^{\prime}$) and mean expression. The red line again depicts the expected relationship under the null hypothesis. **E** Points from panel D colored by their FDR-adjusted *p*-value. Markers are as in panels C-D. **F** Average purity scores (color scale as in panel B) for the dataset shown in panel B for clusters obtained using features selected by BigSur at different *p*-value thresholds. Numbers overlaid on each tile indicate the number of features selected by BigSur in each case.

To guide the use of this model in analyzing real-world data, we first generated simulated data reflective of a situation in which two cell types or states are present, in equal proportions, and differ only in the expression levels of a fixed number of genes. Specifically, for 2,000 cells and 15,000 genes expressed at a range of levels, we generated data using a Poisson log-normal model with an underlying coefficient of variation of gene expression (${c}_{j}$) of 0.7 for each gene (Fig. 3A; see methods). For a subset of genes, the two cell types were made “truly variable”, i.e., different expression means were used for the two cell types, whereas, for the rest of the genome, the means for the two cell types were the same. For simplicity, in Fig. 3 we did not vary the sequencing depth among cells (a common problem in scRNAseq, which we address later on).

When we generate datasets in which the number of truly variable genes is between 100 and 1,000, we observe a threshold above which randomly selected features will enable separation of the two cell types (Fig. 3B). The threshold depends on how many features are truly variable and how many random genes are selected as features, but tends to occur when the number of truly variable genes expected to be found among the random features ranges from 30 to 150 (values along the diagonal in Fig. 3B). As the number of randomly selected features increases, the fraction of truly variable features necessary to separate the two cell types also increases. This agrees with previous results arguing that separating distributions in high-dimensional space depends on including informative features (“signal”) while excluding non-informative features (“noise”) [36].

Ideally, an algorithm for identifying features should seek to compare the observed variance of a feature with the variance expected under the null hypothesis, i.e., when features are not more variable than expected. For data that are Poisson-distributed, variance is equal to the mean, so the ratio of observed variance to mean (also known as the Fano factor) equals 1 under the null hypothesis. Hereinafter we use $\:{\varphi}_{j}$ to stand for the Fano factor of gene $\:{j}$, $\:{\sigma}_{j}^{2}$ the observed variance of gene $\:{j}$, and $\:{\mu}_{j}$ the observed mean. Thus, ${\varphi}_{j}\equiv\frac{{\sigma}_{j}^{2}}{{\mu}_{j}}$.

Figure 3C shows the distribution of observed Fano factors in simulated data generated as in Fig. 3B, in which the number of truly variable genes was 1,000. Although one should only expect to observe $\varphi > 1$ for truly variable genes, many non-variable genes also display large values of $\varphi$; in particular this is true for all of the highly expressed genes. This relationship, previously observed in real data (e.g [19]), reflects the fact that the variance of a Poisson sample from a distribution (hereafter referred to as a Poisson compound distribution) is equal to the variance of the Poisson distribution (i.e., the mean) plus the variance of that distribution. As we may equivalently express variance as the coefficient of variation times the mean squared, i.e., $\:(c{\mu)}^{2}$, a modified Fano factor expressed as:

1 $$\frac{{\sigma}_{j}^{2}}{{\mu}_{j}+({c}_{j}{{\mu}_{j})}^{2}}=\frac{{\sigma}_{j}^{2}}{{\mu}_{j}\left(1+{c}_{j}^{2}{\mu}_{j}\right)}$$

should have an expectation value of 1 for a compound Poisson distribution. Indeed, Fig. 3D shows that, when applied to simulated data, the modified Fano factor is indeed centered around 1 for non-variable genes, and exceeds 1 for variable genes, as long as the latter are sufficiently highly expressed.

Although this modification to the Fano factor clearly facilitates the identification of truly variable genes in the simulated data in Fig. 3, it does not correct for an additional problem encountered in real scRNAseq data, which is that sequencing depth often varies widely (by orders of magnitude) between cells. Simply normalizing observed counts to the total number of reads in each cell is inappropriate—the expected distribution of counts across cells under the null hypothesis is no longer knowable—but sequencing depth variation can be appropriately accounted for by correcting each gene’s Pearson residual [37]. The Pearson residual, a measure of deviation from the mean, is defined, in cell $i$ and gene $\:{j}$, as

2 $${P}_{ij}=\frac{{x}_{ij}-{\mu}_{j}}{\sqrt{{\mu}_{j}}}$$

where ${\mu}_{j}$ is the mean expression over all cells. The Fano factor may in fact be expressed as an average of squared Pearson residuals:

3 $${\varphi}_{j}=\frac{1}{n-1}{\sum}_{i=1}^{n}{{P}_{ij}}^{2}$$

Lause *et al*. noted that, when sequencing depth is variable, the expectation value for ${\varphi}_{j}$ can be made equal to 1 if the term ${\mu}_{j}$ in the Pearson residual of every cell $i$ is replaced by ${\mu}_{ij}$ as follows [37]:

$${\mu}_{ij}=\frac{{\sum}_{j}{x}_{ij}{\sum}_{i}{x}_{ij}}{{\sum}_{ij}{x}_{ij}}$$

  If we further modify (3) by dividing by $\sqrt{\left(1+{c}_{j}^{2}{\mu}_{ij}\right)}$, along the lines described above, we obtain $P^\prime_{ij}$, which we refer to as a “modified corrected Pearson residual”:

4 $$P^\prime_{ij}=\frac{{x}_{ij}-{\mu}_{ij}}{\sqrt{{\mu}_{ij}\left(1+{c}_{j}^{2}{\mu}_{ij}\right)}}$$

as well as a “modified corrected Fano factor”, $\varphi_j^\prime:$

5 $$\varphi_j^\prime=\frac{1}{n-1}{\sum\:}_{i=1}^{n}{P^\prime_{ij}}^{2}$$

Note that $P^\prime_{ij}$ and $\varphi_j^\prime$ are calculated from raw, unnormalized counts, as it is the ${\mu}_{ij}$, and not the ${x}_{ij}$, that undergo correction for sequencing depth variation.

Because we expect $\varphi_j^\prime=1$ under the null hypothesis, observing $\varphi_j^\prime>1$ should identify features likely to be truly variable. To utilize those observations in a principled way, however, requires knowing the probability of observing any value of $\varphi_j^\prime$ under the null hypothesis (i.e., when the cells are all of the same state). While it is generally not possible to obtain an analytical expression for the full distribution of $\varphi_{j}^\prime$, we can estimate it by constructing an arbitrary number of moments of that distribution from the moments of the distributions of the individual $P^\prime_{ij}$, which we can in turn construct from the moments of the Poisson log-normal distribution parametrized by ${\mu}_{ij}$ and $\:{c}_{j}$ in each cell [22]. Given a sufficient number of moments, procedures exist for estimating tail probability densities [38]. In this manner, one can associate any set of gene expression values with a *p*-value, i.e., the probability of observing it by chance. Given a set of *p*-values, one can then choose a *p*-value threshold to control for any level of false discovery [39]. In Fig. 3E, the data displayed in panels C, D are colored by the false discovery rate (FDR) threshold interval into which their *p*-values fall. It is immediately apparent that, for these data, few non-variable genes display FDR-adjusted *p*-values less than 0.05, and none display values less than 0.001.

We have named the algorithm for jointly computing modified corrected Fano factors and their *p*-values *BigSur*, which stands for *B*asic *I*nformatics and *G*ene *S*tatistics from *U*nnormalized *R*eads. Figure 3F applies BigSur to all eight datasets (columns) in Fig. 3B, reporting both the number of features that satisfy different significance thresholds, and their success in clustering the two cell types (the latter represented using the same color scheme as in Fig. 3B). A surprisingly small number of features suffice to produce good clustering (Fig. S5B). For example, in the dataset containing only 100 truly variable genes, as few as 22 statistically significant features recovered pure clusters, whereas no number of random features (from 100 to 15,000) could do so. This reflects the ability of BigSur to recover a large fraction of true positives while minimizing false discovery. Although other feature selection methods, such as HVGs, could also successfully identify the two cell types in these simulated data (Fig. S5), the default number of features selected to use in clustering was often much higher. For example, on the same dataset, at its default dispersion threshold of 0.5, HVGs selected thousands of genes, whereas we found that just using the top 366 of those genes was adequate to produce essentially the same results (Fig. S6).

The only hyperparameter used by BigSur is $\:{c}_{j}$, the underlying coefficient of variation of gene expression (i.e. the actual biological variation among equivalent cells). While this number may, in principle, differ for each gene, experimental studies suggest it does not vary greatly, and we provide a method here to estimate a consensus value of ${c}_{j}$ (which we hereafter simply call $c$) from a full gene expression dataset (see methods).

It is worth noting that the modification and correction of Pearson residuals may also be generalized to higher order statistics, such as correlation coefficients, and that this can be leveraged to more accurately assess gene-gene correlations [22].

### BigSur helps identify rare, biologically relevant cell states

**Fig. 4:**
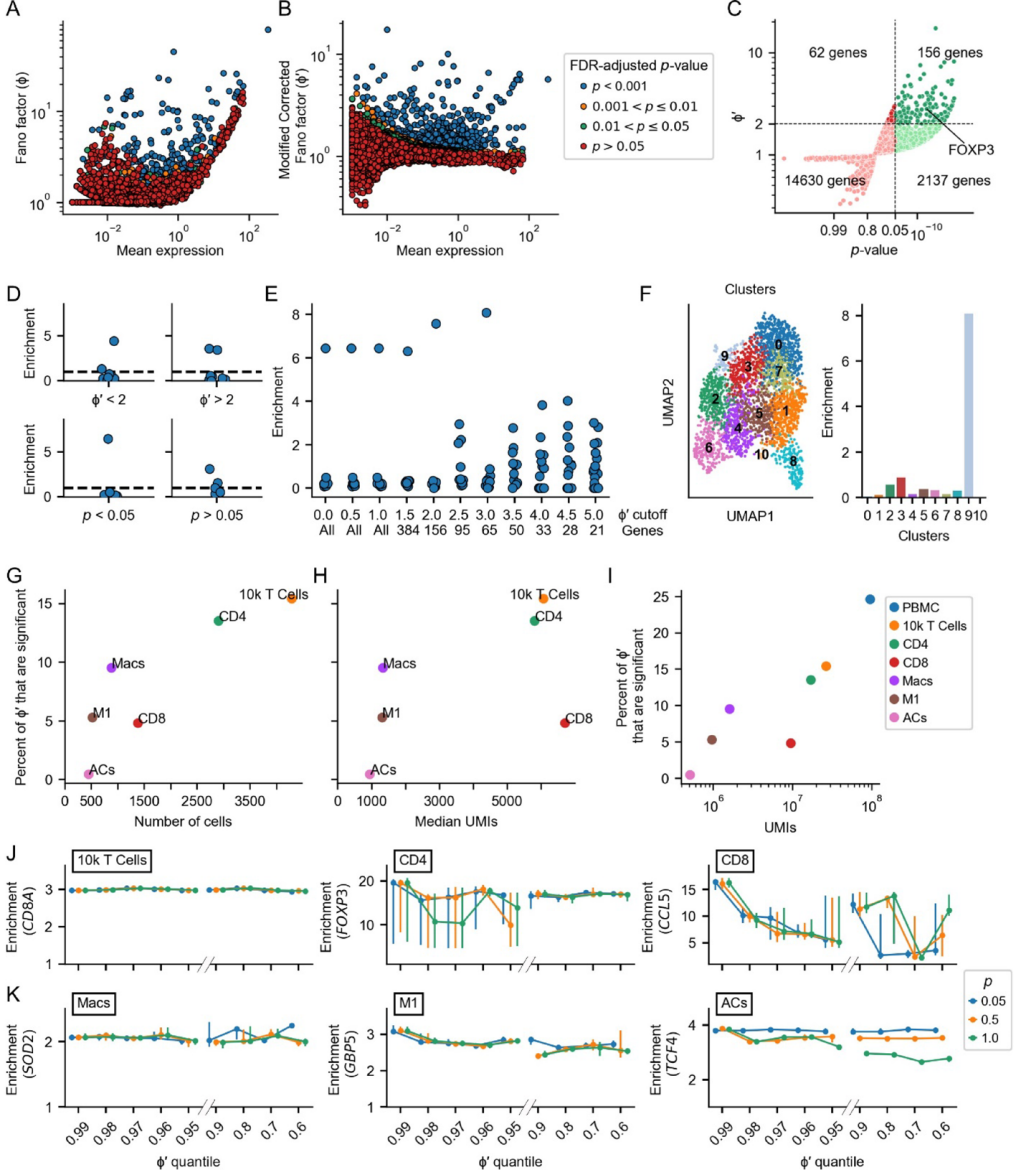
Clustering performance using values and significance levels of modified corrected Fano factors. The Fano factor $\varphi$ (**A**) and the modified corrected Fano factor $\varphi^\prime$ (**B**) were calculated for all genes in the CD4+ T cell dataset and are plotted as a function of gene expression level and colored according to the *p*-value of $\varphi^\prime$. **C** Values of $\varphi^\prime$ and their *p*-values for genes in the CD4 + T cell are plotted against one another. **D–E** Enrichment scores (see main text) for *FOXP3* after Leiden clustering using features selected using different *p*-value and $\varphi^\prime$ cutoffs. Each point represents an individual cluster. Panel D shows the enrichment scores using solely a $\:\varphi^\prime$ threshold (top) or solely an adjusted *p*-value threshold. Panel E displays the enrichment scores as the $\varphi^\prime$ is increased. Only genes with $\:\varphi^\prime$* p*-values < 0.05 were used. **F** UMAP of the CD4 + T cell dataset with genes $\varphi^\prime < 3$ and *p*-value < 0.05 along with each cluster’s F*OXP3 *enrichment (the enrichment of clusters 0 and 10 were 0.04 and 0, respectively). **G–H** For each of six datasets, the percentage of $\varphi^\prime$ that were significant was plotted against the number of cells (panel G) and the median sequencing depth (panel H). **I** for the datasets in panel H, as well as the full PBMC dataset (displayed in Fig. 1), the percentage of $\varphi^\prime$ that are significant was plotted against the total number of transcripts in the dataset. **J–K** The highest enrichment score for six datasets (displayed in panel F) as a function of features selected using different quantile thresholds for $\varphi^\prime$ (abscissa) and different *p*-value cutoffs (by color). The Leiden clustering algorithm was run on the selected features using 40 different randomly chosen starting seeds. For each Leiden seed, the enrichment level in the cluster with the greatest enrichment was found. Each point on the plot indicates the median of this value over the different Leiden seed choices, and the bars show the inter-quartile range (25–75% of the data).

We next examined the performance of BigSur on experimental data, focusing on cases in which the presence of rare cell types, or subtly different cell types, might be expected to pose challenges for clustering. First, we chose the CD4+ T cell subset, which contains previously identified Treg cells, from the 10k PBMC set. As shown in Fig. 4A, the (uncorrected) Fano factors associated with gene expression rose sharply with expression level, similar to what was observed in simulated data (Fig. 3C). In contrast, the modified corrected Fano factors shown in Fig. 4B (using a fitted $\:c$ = 0.25) were generally mean-independent, with most genes displaying a value of $\varphi^\prime$ near 1. These results suggest that the statistical properties of simulated data resemble those of real data. They also suggest that, in this dataset, the patterns of expression of most genes are consistent with not being truly variable. For a small subset of genes, however, values of $\varphi^\prime$ up to 10 were observed, and most of those above 1.5 were associated with FDR-adjusted *p*-values less than 0.05. Overall, 156 genes were characterized by $\varphi^\prime$ >2 and *p* < 0.05 (Fig. 4C). Among these was *FOXP3*, considered a marker for Tregs.

To investigate which statistic, $\varphi^\prime$ or the *p-*value, played a greater role in enabling identification of Tregs as a unique cluster, we carried out Leiden clustering using features identified at different thresholds for these parameters and calculated a *FOXP3*-enrichment score for each cluster produced. The enrichment score was calculated by dividing the mean normalized expression of *FOXP3* in each cluster by the mean expression of *FOXP3* in the dataset. As shown in Fig. 4D, E, use of an adjusted *p*-value threshold of 0.05 was particularly important to achieve good cell separation. The inclusion of non-statistically significant features appears to be especially detrimental to rare cell type identification as Tregs are only 1.8% of the cells in this dataset. Among statistically significant features, a cutoff based on $\varphi^\prime$ appeared to have little impact, until that cutoff became so high that the absolute number of selected features became very small—in the vicinity of 50–150 genes (Fig. 4E). We show the clusters and their enrichment scores when using a $\varphi^\prime$ cutoff of 3 and *p*-value cutoff of 0.05 in Fig. 4F.

We next extended this analysis to five other datasets that contain identified cell “subtypes” (shown in Fig. S7): the full T cell subset of the 10k PBMC dataset, the CD4+ and CD8+ subsets of those T cells, a macrophage dataset, an M1 subset of those macrophages, and the retinal amacrine cell subset. These datasets span a wide range of at least three characteristics: the fraction of all $\varphi^\prime$ observed to be statistically significant (*p* < 0.05), the number of cells sequenced (Fig. 4G), and the median sequencing depth (median UMI/cell) (Fig. 4H). The latter two of these may be expected to have a strong influence on the statistical power to identify differences in cell types—fewer cells mean fewer observations, and lower sequencing depth means more observations that are zero and thus minimally informative. A simple metric that captures a sense of the combined statistical power of these two observations is the total number of UMI in the entire dataset. Plotting this against the fraction of $\varphi^\prime$ values that is statistically significant (Fig. 4I) shows that, overall, the significant $\varphi^\prime$ fraction varies directly with total UMI. This general pattern is to be expected, as loss of statistical power should correlate with identification of fewer $\varphi^\prime$ as significant, however the magnitude of the effect differs between datasets (for example, the fraction of significant $\varphi^\prime$ values is particularly low for the CD8+ T cells). A low fraction of significant $\varphi^\prime$ values, in a dataset containing a sufficient number of well-sequenced cells to provide good statistical power, suggests that gene expression differences between cells are especially subtle in this case.

We next examined how well Leiden clustering using features selected by BigSur performed in enriching for markers of known cell subsets. We used enrichment for *CD8A* to assess clustering of CD8+ T cells from mixed T cells, *FOXP3* for Tregs from CD4+ T cells, *CCL5* for memory T cells from CD8+ T cells, *SOD2* for M1 macrophages from mixed macrophages, *GBP5* for a subset of M1 macrophages, and *TCF4* for glycinergic amacrine cells from total amacrine cells (Fig. S7; see methods). In each case we considered three different FDR-corrected *p*-value thresholds of 0.05, 0.5 and 1.0 and varied the $\varphi^\prime$ cutoff. To facilitate comparisons between datasets, the $\varphi^\prime$ cutoff was varied by quantile, i.e., 0.6 implies the top 40% of $\varphi^\prime$ values, 0.9 the top 10%, and so on. In each case, Leiden clustering was performed 40 times using the features obtained, with a different random seed each time. Shown in Fig. 4J, K are the median and upper and lower quartiles of the enrichment scores obtained from these runs. For the amacrine and T cell datasets, we also performed a full characterization of the effect of using different numbers of BigSur-selected features on the purity score, similar to the analysis done using HVGs in Fig. 2. The results when calculating the purity score and the enrichment score were similar (Fig. S8).  

These outcomes support several of the previous conclusions and suggest guidelines for the use of BigSur in practice. For datasets in which cells are numerous, sequencing is deep, and differences between cell types considerable, clustering succeeds regardless of how features are selected, as in the separation of CD8+ T cells from other T cells or FOXP3+ T cells from other CD4+ T cells, although in the latter case, a too stringent $\varphi^\prime$ quantile cutoff leads to a decrease in the reliability of clustering (Figs. 4E and J and S8F). In such cases, we suggest selecting genes that are statistically significant (*p* < 0.05) and in the top 10% of $\varphi^\prime$. In contrast, for deeply sequenced, large datasets in which fewer than 5% of $\varphi^\prime$ values are statistically significant, such as the CD8+ T cell dataset, we find that restricting feature selection by the relative magnitude and statistical significance of $\varphi^\prime$ can improve performance. For datasets with these characteristics, we suggest only selecting the top 1% of $\varphi^\prime$ that are significant.

In datasets with few cells or shallow sequencing depth, such as the macrophage and amacrine cell datasets, feature selection becomes more challenging. Characteristics of such datasets include having fewer than 150 cells, or less than 3,000 median UMI/cell. In these cases, we suggest selecting the top 10% of the $\varphi^\prime$ that are significant and carefully evaluating the resulting clusters for biological meaning.

### BigSur yields comparable or higher enrichment than other common feature selection methods

We compared BigSur to three of the most common methods for feature selection: HVGs (current default in scanpy), FindVariableFeatures (FvF, default in Seurat V5), and SCTransform v2 (SCT) [19, 23–25]. As mentioned earlier, HVGs ranks features by binning genes by mean normalized expression level, calculating z-scores for the Fano factors of genes with respect to their bin, and ranking the genes by those z-scores [18, 24]. FvF fits the relationship between the logarithm of gene variance and log-mean expression and ranks genes by standardized residuals [23]. SCT fits each gene to a negative binomial model with sequencing depth as an explanatory variable, then regularizes the parameters, and ranks genes by residual variance [19, 25].

**Fig. 5:**
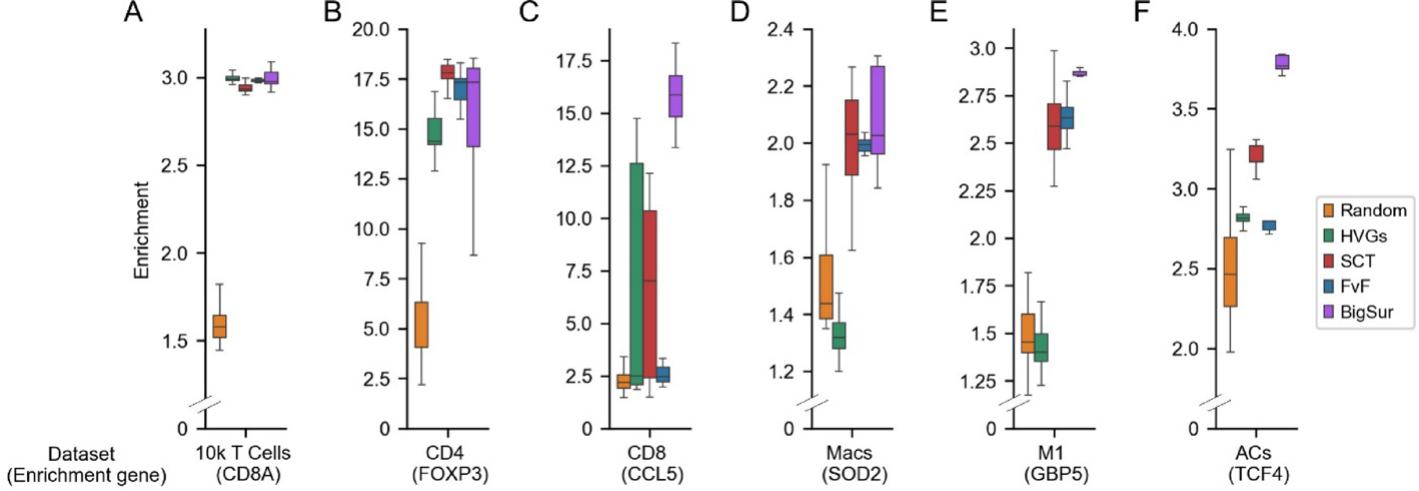
BigSur performs comparably to or outperforms common feature selection methods. Greatest enrichment score (calculated by dividing the mean expression of a gene in each cluster by the mean expression of that gene in the dataset and retaining the greatest value) of six datasets when using different feature selection methods and different Leiden starting seeds. For each of six datasets, features were selected, using either HVGs, FindVariableFeatures (FvF), SCTransform (SCT), BigSur, or a random selection of 2,000 features; clusters were assigned using the Leiden algorithm (with resolution = 1), with 40 different starting seeds; and the highest enrichment score of the gene written in paratheses under each dataset’s name was calculated. HVGs, FvF and SCT selected their default number of genes. BigSur’s cutoffs were set to the top 1% of significant $\varphi^\prime$ for the CD8 dataset, and 10% otherwise.

We compared the performance of each feature selection method using the datasets introduced in Fig. 4. For each dataset, we either randomly selected 2,000 genes, or used genes selected by the other feature selection methods using their default cutoffs. We selected BigSur’s cutoffs using the guidelines described above; consequently, we used the genes with top 1% significant $\varphi^\prime$ for the CD8 dataset, and genes with top 10% significant $\varphi^\prime$ for the other datasets. We then assigned cells to clusters using the Leiden algorithm with 40 unique starting seeds and calculated the enrichment score of the marker gene for each dataset (as in Fig. 4). For the deeply sequenced datasets with many cells and large differences between cells (i.e., the 10k T cells and CD4 datasets), the enrichment yielded by BigSur was equal to, or greater than, any other method (Fig. 5A, B). For the deeply sequenced dataset with many cells and subtle differences between cells (i.e., the CD8 dataset), the median enrichment yielded by BigSur was more than twice than that of the next best method (SCT), and the median enrichments of other methods performed similarly to random feature selection (Fig. 5C). For the three shallowly sequenced datasets (i.e., the two macrophage datasets and the amacrine cells dataset), BigSur again yielded comparable or higher enrichment than the enrichments produced by other methods (Fig. 5D–F).

These results suggest that BigSur’s feature ranking and the cutoffs chosen for each dataset are comparable to, if not better than, those of commonly used methods.

### Comparative performance of BigSur on datasets with known labels

The data in Fig. 5 assess the ability of various feature selection methods to enrich for genes known to mark specific cell subtypes. An alternative method for comparing performance is to use datasets in which ground truth cell type labels are known *a priori*, comparing the ability of unsupervised methods to cluster them correctly. To create experimental data in which ground truth labels are known, yet are also sufficiently challenging to be sensitive to the choice of feature selection method, we generated datasets consisting of pairs of groups of cells (generally ones that could be identified by any of the features selection algorithms) and downsampled one of the groups to make the task of discriminating between them more challenging.

We started with four datasets—a 1 million cell (1M) PBMC dataset, a skin dataset [40], the 10k PBMC dataset introduced in Fig. 1, and the macrophage dataset introduced in Fig. 4. Each was subsetted to contain only two groups of related cells (i.e., cells that display common cell type specific markers; see Fig. S9 and methods). Initially, ground truth labels corresponded to cluster identities found using features selected by BigSur; however, similar clusters could be obtained using any of the feature selection methods, using their default parameters, in almost every case (the one exception being that HVGs was unable to separate M1 and M2 macrophages; Figure S10). Interestingly, this was observed despite the fact that the rank-orders of features produced by different methods were quite different; indeed, all feature selection methods highly ranked some genes that BigSur considered to be non-significant (Fig. S11). We then randomly selected a small homogenous population of cells from one of the two groups (the “rare” cells) and a larger homogenous population from the other group (the “common” cells), and combined them so that the rare cells made up 5% of the total. The steps in generating these “semi-synthetic” datasets are outlined in Fig. S12 and described in methods. Because sampling was random, the process was replicated 20 times, generating 20 semi-synthetic datasets from each of the four datasets.

**Fig. 6:**
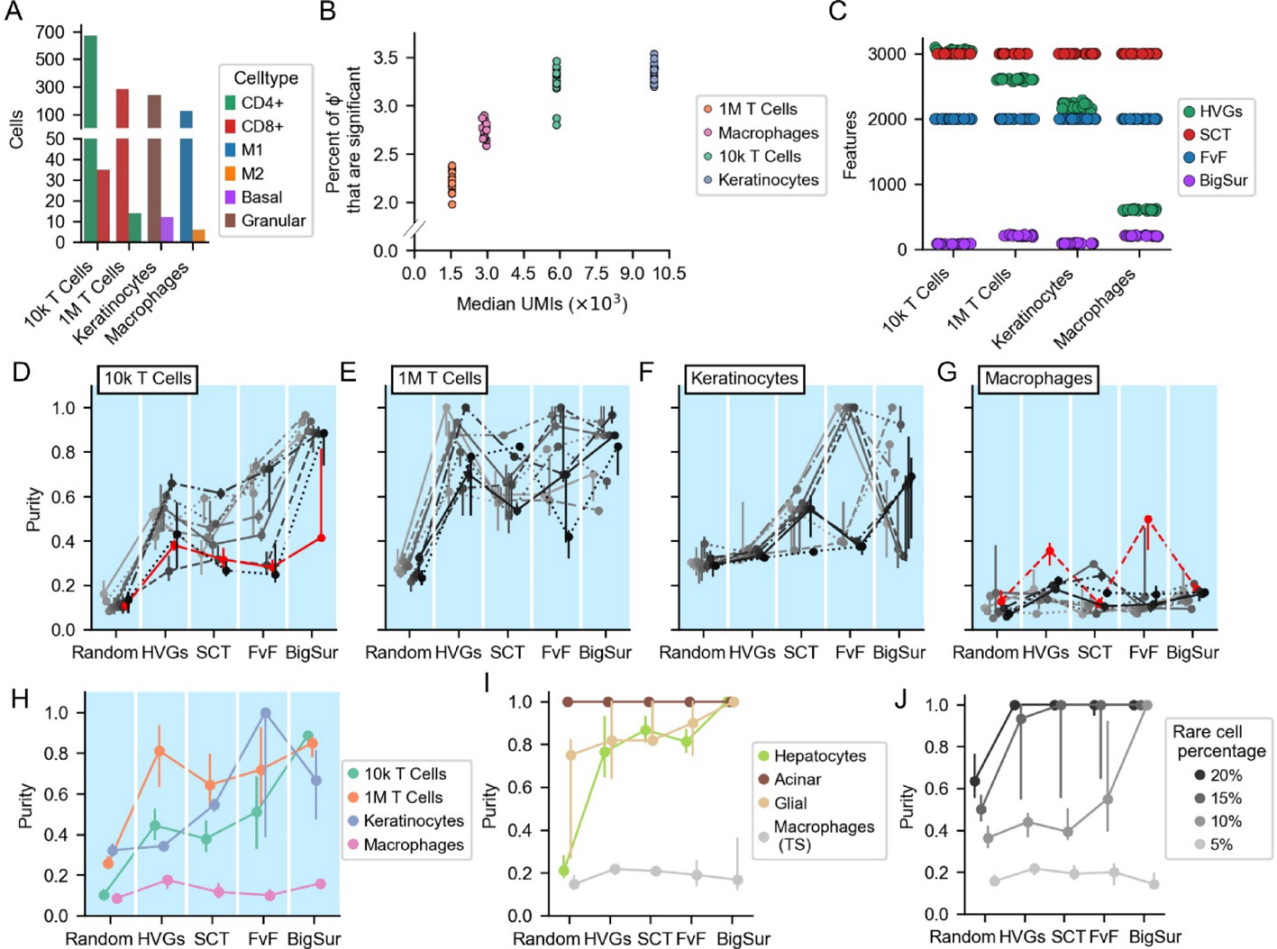
BigSur improves clustering performance using fewer total features. For each of four datasets (two PBMC datasets, a skin dataset and a macrophage dataset, see main text), 20 “semi-synthetic” datasets were generated (see methods). **A** Bars show the cell numbers for each cell type in each semi-synthetic dataset. **B** The percent of $\varphi^\prime$ values with *p* < 0.05 is plotted as a function of the median UMI/cell per dataset. **C** The number of features selected by each method for each semi-synthetic dataset (see main text for BigSur’s cutoffs). **D–H** Purity scores of each semi-synthetic dataset as the feature selection method was changed. For each of the semi-synthetic datasets, Leiden clustering, using 40 different random starting seeds, was performed using features that were either a random selection of 2,000 genes, or those chosen by HVGs, SCT, FvF and BigSur. Purity scores were calculated (as in Fig. 2). Purity scores of semi-synthetic datasets generated from the 10k T cell, 1M T cell, keratinocyte and macrophage datasets are shown in panels D, E, F and G respectively. Each line represents a different semi-synthetic dataset (for clarity, only 10 of the 20 are shown in each plot; see Fig. S12 for the remaining data), and the markers and bars are the medians and interquartile ranges (IQRs) of the purities (over the 40 different Leiden seeds). The purities from selected semi-synthetic datasets are colored for discussion purposes (see main text). **H** Median purities of semi-synthetic datasets. The markers represent medians and the bars represent IQRs. **I** Median purities of 20 semi-synthetic datasets generated from four datasets included in the Tabula Sapiens (TS) human cell atlas (see main text). Purities were calculated using the same procedure as for the data shown in panel H. **J** Purity scores of semi-synthetic datasets generated from the macrophage (TS) dataset with varying percentage of rare to total cells, holding the number of total cells constant. 20 semi-synthetic datasets were generated for each percentage.

The total numbers of cells in these semi-synthetic datasets ranged from 132 to 705 (Fig. 6A). The percentages of $\:{\varphi\:}^{{\prime\:}}$ values that were judged significant (*p* < 0.05) was relatively low in all cases, around 3%, and median total UMI varied between about 1,500 and 10,500 (Fig. 6B). Given the results in Fig. 4, we expected these conditions would make it relatively difficult to identify the rare cell type in these cases.

For each of the 80 semi-synthetic datasets, we ran the three common feature selection methods (using default parameters) and BigSur. In each case, we also randomly chose a single set of 2,000 genes to serve as random features.

The default number of features selected by the different algorithms is shown in Fig. 6C. SCT and FvF uniformly chose 3,000 and 2,000 features, respectively. The number of features HVGs chose varied between a high of 3,094 for the 10k T cell semi-synthetic datasets and a low of 589 for the macrophage semi-synthetic datasets. Thresholds for feature selection using BigSur were informed by the results in Fig. 4, as described above. Since the 10k T cell and keratinocyte semi-synthetic datasets had relatively many cells (more than 300), displayed high median UMI/cell (around 5,870 and 9,900, respectively), and each had less than 5% of $\:{\varphi\:}^{{\prime\:}}$ that were significant, we used a $\:{\varphi\:}^{{\prime\:}}$ quantile cutoff of 0.99. Because the 1M T cell and macrophage semi-synthetic datasets both have low median UMI/cell (1,561 and 2,910 UMI/cell, respectively) as well as a low numbers of cells (298 and 132), we used a more generous $\varphi^\prime$ quantile cutoff of 0.9. The number of features selected by BigSur ranged from 79 to 227, far fewer features than those selected by any other method.

We then used the selected features for clustering each of the 80 semi-synthetic datasets. As before, we used 40 independent starting seeds for Leiden clustering. We assessed performance in correctly isolating the rare cells using the same purity score as was used in Fig. 2 (fraction of rare cells in the cluster with the most rare cells). For each of the four datasets, we show results from 10 representative semi-synthetic datasets in Fig. 6D and G, presenting the remaining 10 in Fig. S12E. Figure 6H summarizes the findings of Fig. 6D and G, showing the median and interquartile range of purities obtained using each feature selection method.

For the 10k T cell datasets (Fig. 6D), clustering using random features yielded purities ranging from 0.08 to 0.16. Using HVGs and SCT, purities ranged from 0.26 to 0.66 and 0.27 to 0.62, respectively. FvF yielded a wider range of purities, from 0.25 to 0.74. With BigSur, purities were higher than 0.8, with the exception of a single semi-synthetic dataset (colored in red, purity of 0.42), however this dataset was particularly sensitive to Leiden starting seed, and for many starting seeds BigSur again produced very high purity. Overall, the average performance of features selected by BigSur was higher than with any other method (Fig. 6H).

For the 1M T cell datasets (Fig. 6E), random feature selection yielded purities ranging from 0.22 to 0.33, whereas HVGs, SCT, FvF and BigSur produced similar maximal purities (1.0, 0.88, 1.0 and 0.97 respectively), albeit with considerable variability. Overall, BigSur produced the highest median purity (Fig. 6H).

With the keratinocyte datasets, random feature selection and HVGs both performed poorly, yielding purities spanning 0.28 to 0.39 and 0.32 to 0.36 respectively (Fig. 6F). The performances of SCT, FvF, and BigSur were all highly variable, ranging from poor to very good depending on the dataset. The median performance of FvF exceeded that of BigSur but the range of purities yielded by FvF was greater than that of BigSur (Fig. 6H).

Finally, with the macrophage datasets, all methods yielded very poor purities, although an occasional dataset displayed slightly better purity when using either HVGs or FvF (Fig. 6G, colored in red).

Overall, even though no feature selection method consistently outperformed in all datasets, BigSur was the only method that had median scores above 0.6 for the first three datasets (Fig. 6H). While the median purity yielded by FvF was larger than that of BigSur on one dataset, the purity scores FvF produced in the 10k and 1M T cell datasets and the keratinocyte datasets were more widely spread than those of other methods, suggesting that FvF was overly sensitive to the specific cells composing each semi-synthetic dataset.

Taken together, these results argue that the performance of BigSur is generally as good as or better than other feature selection methods.

### BigSur also outperforms other methods in less challenging datasets

The semi-synthetic datasets described in the previous section not only contained a rare cell type, but many contained relatively small numbers of cells as well as relatively low values of UMI per cell (Fig. 6A, B). Such characteristics would be expected to make feature selection especially challenging. To see whether BigSur continued to provide advantages with less challenging datasets, we generated additional semi-synthetic datasets in which sequencing depth was higher. For this purpose, we downloaded four datasets from the Tabula Sapiens (TS) human cell atlas (liver, pancreas, eye, and lymph nodes) [41]. For each, we selected a particular cell type (hepatocytes from the liver, acinar cells from the pancreas, Müller glial cells from the retina, and macrophages from the lymph node), clustered it, and selected two subclusters (see Fig. S13, procedure detailed in methods) which were subsequently used to generate semi-synthetic datasets as described above (Fig. S14A–D). Figure S14E, F shows the number of cells and statistics of these datasets. Dataset sizes varied from ~100 to ~250 cells, and median sequencing depth ranged from 9,136 to 31,997 UMI/cell.

Using each dataset, we selected features, assigned clusters using 40 different Leiden starting seeds, and calculated purity scores. We selected BigSur’s cutoffs by following the previously stated guidelines: for the hepatocyte and acinar semi-synthetic datasets, we selected genes with the top 1% of significant $\varphi^\prime$, and for the macrophage (TS) and glial semi-synthetic datasets, we selected genes with the top 10% of significant $\varphi^\prime$.

All methods performed poorly in the macrophage (TS) semi-synthetic datasets and performed nearly perfectly in the acinar datasets; as for the other datasets, only BigSur yielded median and 25th quantiles equal to unity (Fig. 6I). The macrophage (TS) semi-synthetic datasets only contained 5 rare cells, out of a total of 114 cells; we therefore generated additional macrophage (TS) semi-synthetic datasets with increasing percentages of rare to total cells, keeping the total number of cells at 114. When increasing the percent of rare cells to 10%, an increase of only 5 cells, the median purity using BigSur increased to unity, whereas the other methods generally performed similarly to random feature selection (FvF did slightly better; Fig. 6J). As the ratio of rare to total cells was increased, the purity scores yielded by all methods increased, and all methods except FvF had median and 25th quantile scores of unity.

These results suggest that in small, deeply sequenced datasets with few rare cells, BigSur still outperforms common feature selection methods.

### Validation of semi-synthetic results

In comparing the performance of different feature selection methods, a variety of choices had to be made regarding, for example, the metric used to assess performance, the resolution used during clustering, the number of genes selected by each method, the size of the semi-synthetic datasets, and how semi-synthetic datasets were generated. To ascertain whether any of these choices might have inadvertently biased results in favor of BigSur, we explored a variety of alternative choices. As summarized below, in each case, we did not observe substantive changes in qualitative conclusions.

First, we considered two alternatives to purity scores: we either replaced purity scores with their calculated *p*-values (i.e., their probability of occurring by chance under the assumption that target cells are evenly distributed among all clusters), or we replaced purity scores with an alternative measure of diversity, the normalized entropy [42]. When calculating these metrics for the 10k T cell, 1 MT cell, keratinocyte, and macrophage datasets, we found that normalized entropy and purity score had similar trends, and all *p*-values of the purity scores–besides those produced in the macrophage semi-synthetic datasets–were lower than 0.05 (Fig. S15A, B).

Next, we examined the effect of the resolution parameter used during Leiden clustering, which influences the number of clusters that are identified. We varied Leiden resolution in each semi-synthetic dataset generated from the 10k T cell dataset and calculated purity scores [33]. We found that the purity scores from BigSur selected features were consistently higher than those from any other selection method at the same resolution (Fig. S15C-D). Interestingly, we also found that the number of clusters yielded by BigSur tended to increase more slowly with increasing resolution than other methods (Fig. S15E).

We next asked whether reducing the number of features selected by HVGs, FvF and SCT from their default values might improve purity scores in the 10k T cell, 1M T cell, keratinocyte, and macrophage semi-synthetic datasets (in effect testing whether these methods were, in fact, ranking features well, but simply choosing too many of them). We thus lowered the number of features selected by each method to the number of features that BigSur had selected, assigned clusters, and calculated purity scores. The results were mixed: only the purities yielded by SCT improved for all datasets. With HVGs and FvF, some purities increased and others decreased (Fig. S16A). In a converse experiment, we increased the number of genes selected by BigSur in semi-synthetic datasets with 10% rare-to-total cells (shown in Fig. 6I) and found that median purities fell from 1 to 0.45 (Fig. S16B).

We also investigated how important the number of cells in a dataset was in influencing the difficulty of feature selection. To do this, we generated additional semi-synthetic datasets with greater numbers of cells (numbers of cells and statistics of the datasets shown in Fig. S17A, B; see methods for details) and found that most feature selection methods yielded purities of one, with the exception of FvF which performed similarly to random feature selection in the macrophage (TS) semi-synthetic datasets (Fig. S17C).

Finally, since we had used BigSur to initially identify the cell types used in creating semi-synthetic datasets, we considered the possibility that doing so might have unfairly advantaged BigSur in performing the task of clustering the two cell types. To test this, we generated additional semi-synthetic datasets, using HVGs to select features at each subsetting step and during the semi-synthetic generation process (the intermediate steps for doing so are outlined in Figs. S18, 19 and detailed in the methods). The statistics of these semi-synthetic datasets are shown in Figure S20A, B. We found that BigSur still yielded the highest or second highest purities for all semi-synthetic datasets, except for those generated from the 10k T cell datasets (Fig. S20C, D). Interestingly, while subsetting the pancreas dataset, we noticed that two clusters with high expression of an acinar marker (*CLPS*) defined using features produced by HVGs had an order-of-magnitude difference in sequencing depth (clusters 0 and 1, shown in Fig. S20E), suggesting that artifacts reflective of sequencing depth differences might have driven the clustering of these cells into two distinct groups. Consistent with this view, BigSur reported that nearly all $\varphi^\prime$ in datasets produced by mixing these clusters were statistically insignificant, and therefore did not justify subdivision into two separate clusters.

Collectively, these results strengthen our conclusions that BigSur performs as well or better than other methods in a wide range of datasets.

### Comparison of speed and memory usage of different feature selection methods

We compared the speed of computation of each feature selection method on datasets of varying size. To do so, we generated datasets with increasing numbers of cells by randomly sampling from the full 1M PBMC dataset and calculated the speed of each method for each set of cells (see methods). BigSur was slower than HVGs and FvF but faster than SCT (Fig. S21A). At least 90% of the computation time for BigSur appears to be spent on *p*-value calculation (compare dashed line in Fig. S21A). Even with 100,000 cells, however, BigSur finishes in about a minute and a half.

We also wanted to measure the speed of computation relative to the number of genes. Accordingly, we generated datasets from the 1M PBMC dataset with 10,000 cells and varying number of genes. SCT was the slowest method overall, followed by BigSur (with and without *p*-value calculations), FvF and HVGs (Fig. S21B).

We also measured the total memory used by each method on datasets of varying cell and gene numbers. When varying numbers of cells, HVGs and BigSur’s memory usage stayed relatively small, up to 0.59 GB and 0.97 GB respectively when each method was run on a dataset of 250,000 cells (Fig. S21C). The memory usage of FvF on a dataset of the same size was 9.5 GB. We were unable to run SCT on a dataset of this size, due to insufficient memory; the total memory usage of SCT increased substantially with increasing number of cells, up to 815 GB with 100,000 cells. When varying the number of genes, SCT was also the most memory intensive method, requiring up to 49 GB in a dataset with 10,000 cells and 21,819 genes (Fig. S21D). In that dataset, the memory usages of the other methods were similar to one another, at around 0.86 GB.

## Discussion

Feature selection is known to be an important general step in machine learning [9], but the impact of different methods of feature selection on single cell transcriptomics has not been fully explored. Currently, several methods are in common use as part of popular analysis packages, and multiple others have been proposed as potential improvements [10–19]. Clear guidance about how to choose among them, or when to consider adjusting their parameters, is difficult to come by. Although some head-to-head comparisons have been published (e.g [13, 20, 21]), attention is not often paid to the difficulty of the task for which feature selection is used. Here, we focus on unsupervised cell clustering and note that, in many cases–such as when cells are highly distinct in gene expression–feature selection hardly matters, and random sets of genes often do nearly as well as carefully chosen ones. Under these circumstances, even if large differences exist in the accuracy with which different methods identify truly variable genes (e.g [12]), they are likely to be of little practical importance.

In contrast, it is when attempting to separate subtly dissimilar groups of cells, especially when present as minor cell subpopulations, that the choice of feature selection method and the number of selected features can greatly impact accuracy of clustering. The impact of feature selection in this regime has been relatively unexplored, even though it may be expected to arise in many common applications, such as when repetitively subclustering cell populations; characterizing cell heterogeneity in tumor samples; or distinguishing intermediate states in cell lineages.

The results presented here argue that, in such cases, it is important to use feature selection methods that not only identify genes that are truly variable but also eliminate ones that are not. Ultimately, the success of clustering must reflect a balance between signal (true positives) and noise (false positives). When the proportion of true positives is high, contamination by false positives may be irrelevant, but at lower signal strengths eliminating false positives is essential.

Here we developed a straightforward analytical approach, BigSur, that models the null distribution of gene expression observations as Poisson random variates from a distribution reflecting biological gene expression noise, the coefficient of variation of which is estimated from the data. For each gene, BigSur returns both a measure of variability $\:{\varphi\:}^{{\prime\:}}$, and—by modeling the gene expression noise distribution as log-normal—the probability of observing that value by chance. The method automatically accounts for differences in data sparsity across genes, and differential sequencing depth across cells, so no data normalization or transformation is required. Whereas other feature selection methods sometimes work by fitting data to distributions, those distributions are typically inferred from empirical observations of scRNAseq datasets (e.g [19, 23]), whereas with BigSur the use of the lognormal distribution to represent gene expression variability is grounded in both theory and observation.

We showed here that BigSur can select informative genes and eliminate non-informative ones in both simulated and real datasets and described how simple dataset statistics can be used to limit the number of selected genes. We then compared the performance of BigSur against three popular feature selection methods: FindVariableFeatures (the default in Seurat), HVGs (the default in scanpy), and SCTransform v2 [19, 23–25]. Both simulated data, real data, and “semi-synthetic” datasets formed by guided subsetting of real data were used, concentrating on what we expected to be especially challenging regimes. Not only did these three methods highly rank genes considered non-significant by BigSur, they tended to deliver much longer lists of features. For the most part, BigSur performed as well as or better than other algorithms (Figs. 5 and 6), despite using far fewer features, suggesting that, overall, it has a substantially better signal-to-noise profile.

We did not assess the ability of BigSur to assist tasks other than cell clustering, such as in pseudotemporal ordering, but suspect that the improvement in signal-to-noise ratio in feature selection will be helpful in that setting as well. Also, as noted above, the modified corrected Pearson residuals produced by BigSur may be used to generate modified corrected Pearson correlation coefficients, from which meaningful networks of gene co-expression correlation may be inferred, thanks to a dramatic reduction in false positive correlation [22].

## Conclusions

We show that the importance of feature selection algorithm–how features are ranked and the number of features selected–is related to the difficulty of the clustering task. From simple assumptions, we derive a statistically principled model, BigSur, that accounts for biological and technical noise in single cell transcriptomics. We show how summary statistics calculated using BigSur, along with summary statistics of the dataset, can inform feature selection. Careful consideration of feature selection in single cell transcriptomics analyses improves clustering accuracy, which will in turn improve the quality of results from subsequent tasks.

## Methods

### The modified corrected Fano factor $\varphi^\prime$

The Fano factor quantifies how much the variance of a distribution differs from that of a Poisson distribution. To the extent that scRNAseq data are not generally Poisson-distributed, the Fano factor does not reliably measure unexpected variability, but it can be modified, given an appropriate model, to do so. For any gene, assuming the null hypothesis (i.e, all cells draw transcripts from the same distribution), we model scRNAseq data as a random Poisson sample from a log-normal distribution, the mean of which is scaled in each cell by that cell’s sequencing depth.

To account for variance of sequencing depth across cells, we follow [37] in correcting Pearson residuals. The Pearson residual is a measure of each observation’s difference from the mean, scaled to the square root of the mean (Eq. (2), Results section). We correct it by using a different definition of mean in each cell, one that is scaled to account for total number of UMI in the cell.

The corrected Pearson residual is then further modified to account for the expected greater variance of a compound Poisson log-normal distribution, rather than that of a Poisson distribution. The variance of a compound distribution (assuming independence) is the sum of variances of the two underlying distributions. If the first is a Poisson distribution, then its variance is equal to its mean. For the second distribution, we equivalently express variance as $\:{c}^{2}{\mu}^{2}$, the square of the coefficient of variation times the mean. The total variance is therefore ${\sigma}^{2}=\mu+{c}^{2}{\mu}^{2}=\mu(1+c^2\mu)$. We then use the square root of this expression to replace the denominator of the corrected Pearson residual. Introducing indices and to represent cell and gene, respectively, we obtain the following modified corrected Pearson residual ${P}_{ij}^\prime$:

$$P_{ij}^\prime=\frac{x_{ij}-{\mu}_{ij}}{\sqrt{{\mu}_{ij}\left(1+{c}^{2}{\mu}_{ij}\right)}}$$

Just as the Fano factor, for gene ${j}$, may be expressed as an average over squared Pearson residuals, so may the modified corrected Fano factor be defined as

$$\varphi_j^\prime=\frac{1}{n-1}\sum_{i=1}^{n}{P^\prime_{ij}}^{2}=\frac{1}{n-1}\sum_{i=1}^{n}\frac{{\left({x}_{ij}-{\mu}_{ij}\right)}^{2}}{\mu_{ij}\left(1+{c}^{2}{\mu}_{ij}\right)}$$.

Where $n$ is the number of cells. Note that the modified corrected Pearson residual can also be used to derive other useful statistics, such as a modified corrected Pearson correlation coefficient [22].

The only hyperparameter used in these calculations is $c$, which may be estimated from the behavior of an entire dataset, as described below. Note that the expectation value of $\:{\varphi}_{j}^\prime$ under the null hypothesis is unity regardless of the underlying distribution that describes gene expression. The choice of the log-normal distribution here only influences the expectation value for higher moments of $\:{P}^{{\prime}}$, which are used in the calculation of *p*-values.

### Calculation of $\varphi_j^\prime$* p*-values  

The analytical nature of the model underlying the calculation of $\varphi_{j}^\prime$ allows one to estimate, given a set of observations and cell sequencing depths, the probability that any given value of $\:{\varphi\:}_{j}^{{\prime\:}}$ would arise by chance. Specifically, for each gene, we calculate the moments of the Poisson log-normal distribution [43], from which we calculate the moments of the modified corrected Pearson residuals, which in turn are used to calculate the first 5 moments of $\:{\varphi\:}_{j}^{{\prime\:}}$. These moments describe the distribution of $\:{\varphi\:}_{j}^{{\prime\:}}$ under the null hypothesis, which is that gene $j$ is measured in a homogenous group of cells. We then use numerical procedures [38] to estimate the quantile of an observed $\:\varphi_{j}^\prime$ in this distribution, which may be expressed as a *p*-value. Derivation of the equations for the moment calculations may be found in the appendix of [22].

### Fitting a coefficient of variation for underlying gene expression

Under the assumptions that $c$ is approximately equal across genes, and that most of the genes in a dataset are not significantly differentially expressed across cells, one can estimate by finding the value that minimizes the difference between $\varphi_{j}^\prime$ and 1, for the majority of genes. Because influences the modified corrected Pearson residual only through the term $\:1+{c}^{2}{\mu}_{ij}$, it follows that the choice of $c$ has little influence on $\varphi_{j}^\prime$ when $\:{\mu}_{ij} << 1$. We learn by finding the value that minimizes the absolute value of the slope of a linear fit to a plot of $log_{10}(\varphi_{j}^\prime)$ vs. $log_{10}(\mu_j)$, for ${\mu}_{j}\in\left[0.01,10\right]$.

### Generation of simulated datasets

We first generate underlying means for each gene by sampling from a log-uniform distribution:

$$x \sim (-1, 0.8)$$

$$\mu_{j,k} = 10^x$$

Where ${\mu}_{j,k}$ is the mean of gene ${j}\:$ for cell type $k$. We then generate transcript counts for each cell by sampling from a log-normal distribution with ${\text{c}}\:=\:0.7\:{:}$

$$y_{{i,j,k}} \sim \textit{Log-Normal}(\mu _{{j,k}},c) $$

Where $\:{y}_{i,j,k}$ is the count of gene $j$ in cell $i$ of cell type $k$. Note that in many texts, log-normal distributions are parametrized in terms of the mean and standard deviation of the underlying normal distribution from which they may be derived, but here $\:{\mu}_{j,k}$ and $c$ refer to the actual mean and coefficient of variation of the log-normal distribution.    

Finally, we use $\:{y}_{i,j,k}$ as the rate parameter for a Poisson distribution and sample from it to simulate the sequencing of transcripts from each cell:

$$z_{{i,j,k}} \sim {Poisson}\left( {y_{{i,j,k}} } \right) $$

Where ${z}_{i,j,k}$ is the simulated UMI for each gene in each cell. The datasets in Fig. 3 simulate 2,000 cells, each expressing 15,000 genes. The cells are divided into two equally sized groups of 1,000. For some of the genes (“not truly variable” genes), expression values for both groups are generated using a single ${\mu}_{j,k}$. For other genes (“truly variable” genes), the $\:{\mu}_{j,k}\:$in the two groups of cells are generated using independent, random samples of $\:x$ (i.e., ${\mu}_{j,k=1}\ne{\mu}_{j,k=2}$). Thus, the degree to which “truly variable” genes differ in expression between the two groups of cells is itself distributed about a mean of zero.

For the simulated data, clusters were assigned using the Leiden algorithm with a resolution parameter of 0.1. In Fig. 3B, ten rounds of random feature selection and Leiden clustering were done on each dataset and the average results across the ten trials is presented. In Fig. 3F, Leiden clustering was performed 50 times using unique random seeds and results were averaged across all trials.

### Dimensionality reduction and clustering

Dimensionality reduction and clustering were done using scanpy v1.8.2. Counts were log-normalized as follows:

$${x}_{i,j}=ln\left(\frac{{counts}_{ij}}{\sum_i counts_{ij}}*{10}^{4}+1\right)$$

Where ${x}_{i,j}$ is the log-normalized count of gene $\:j$ in cell $i$.

Once features were selected, regardless of feature selection method, principal component analysis (PCA) was performed on the log-normalized data and the top 50 principal components (PCs) were retained. The *k*-nearest neighbors graph was calculated from the PCs, and UMAP dimensions were calculated using scanpy default parameters. Clusters were identified using the Leiden algorithm implemented by scanpy with default parameters and, if not otherwise stated, resolution = 1 and random seed = 0 (default).

### Classification of cell types using random genes

Cell types in the 10k PBMC dataset were identified using marker genes on clusters calculated as specified above, using HVGs with defaults as feature selection. scrublet v0.2.3 [44] was used to identify doublets using default parameters. For every set of randomly selected genes (selected by uniform sampling using numpy.default_rng.choice function, setting the replace parameter to False [45]), PCA was calculated using sklearn’s [46] implementation of TruncatedSVD with number of PCs = 50, except for 25 genes where number of PCs = 25. A linear support vector machine (SVM, using sklearn.svm.SVC) was trained on each set of PCs calculated by TruncatedSVD, with ground truth labels being the cell types identified as described above. The adjusted Rand index (ARI) and normalized mutual information (NMI) scores were calculated by comparing predicted labels of the SVM to ground truth labels, using sklearn’s implementation of both scores.

### Differential expression testing

To test for differential gene expression (as in Fig. S4), for a given clustered dataset, we compared the log-normalized expression of each gene in each cluster to the log-normalized expression of that gene in the rest of the dataset, using the Mann-Whitney-U test (MWU) [47]. We corrected the *p*-values for multiple testing using the Benjamini-Hochberg approach [39].

### Generation of semi-synthetic datasets

To compare feature selection methods, we devised a procedure that creates datasets in which there are two populations of relatively similar but transcriptionally distinct cells, one substantially rarer than the other. The procedure entails taking pairs of transcriptomically similar groups of cells that had previously been labeled, clustering each group to identify homogenous populations, and mixing small numbers of cells from these homogenous populations together. We detail the identification of the pairs of groups of cells belonging to each dataset in the next three sections.

Specifically, for a given pair of groups of cells, we clustered them separately, by selecting genes using either BigSur or HVGs and clustering using the Leiden algorithm with resolution = 1. For the semi-synthetic datasets analyzed in Fig. 6A–I (i.e., the 10k T cell, 1M T cell, keratinocyte and macrophage semi-synthetic datasets, along with the “smaller” TS semi-synthetic datasets), we selected the largest cluster from each group. We then randomly downsampled the smaller of the selected clusters, and combined it with the larger cluster, such that the resulting ratio of cells from the smaller and larger cluster was 1:19.

For the datasets analyzed in Fig. 6J, i.e., the semi-synthetic datasets with varying percentages of rare cells, we started with the same clusters used to generate the macrophage (TS) semi-synthetic datasets and adjusted the ratio of cells sampled from each cluster, holding the number of cells constant.

For the datasets analyzed in Fig. S16H–J, (i.e., the “larger” TS semi-synthetic datasets), we clustered the smaller of the two groups (resolution = 1) and selected the largest of the resulting clusters. We subsequently randomly sampled cells from the larger of the two groups, without clustering, and combined the sampled cells with the selected cluster, such that the ratio was 19:1 common to rare cells.

To generate the macrophage, glial and macrophage (TS) semi-synthetic datasets using HVGs, we clustered each group using a resolution = 0.5, selected the largest cluster from each group and randomly downsampled the smaller of the selected clusters, and combined it with the larger cluster, such that the resulting ratio of cells from the smaller and larger cluster was 1:19.

For each semi-synthetic dataset, we removed genes expressed in fewer than three cells before selecting features. The cell barcodes of each semi-synthetic dataset were saved (see data availability).

### Subsetting of the 10k T cell, 1M T cell, macrophage and keratinocyte datasets to make semi-synthetic datasets

To make semi-synthetic datasets, we first subsetted each dataset to only include two groups of cells. We used the Leiden algorithm to cluster, with resolution = 1 if not otherwise specified.

For the 10k PBMC dataset, we removed all cells except for the CD4+ and CD8+ T cells, which were previously identified (see Figs. S1 and S9A, B).

For the 1M PBMC dataset, due to the large numbers of cells in this dataset, we did several rounds of downsampling. We started by removed all cells with no expression of *CD3E*, and subsequently removed all genes expressed in less than 3 cells and removed all cells expressing less than 400 genes. We then selected features using BigSur (only selecting the top 2% of ${\varphi}^{{\prime}}$, without considering *p*-value) and clustered on the top 50 PCs. This procedure identified 9 clusters (Fig. S9C). We then removed clusters 6 and 8, removed all genes expressed in less than 3 cells and removed all cells expressing less than 400 genes, and selected features using BigSur (only selecting the top 2% of $\:{\varphi\:}^{{\prime\:}}$, without considering *p*-value), and clustered, which identified 11 clusters (Fig. S9D). We removed all cells except for those in clusters 0 and 3, removed genes expressed in less than 3 cells, selected features using BigSur (selecting the top 10% of significant $\:{\varphi\:}^{{\prime\:}}$) and clustered, which identified 7 clusters (Fig. S9E). From these clusters, we selected clusters 3 and 4, removed genes expressed in less than 3 cells, selected features using BigSur (selecting the top 10% of significant ${\varphi}^{{\prime}}$) and clustered, which yielded 6 clusters (Fig. S9F). All cells except for clusters 1 and 3 were selected, and we identified the CD4+ and CD8+ T cells using *CD4* and *CD8* (Fig. S9G, H).

For the macrophage dataset, we removed all genes expressed in less than 3 cells and removed all cells expressing less than 400 genes. We subsequently removed the unstimulated macrophage progenitor cells, annotated as “BM0” cells, removed genes expressed in less than 3 cells, selected features using BigSur (selecting the top 10% of ${\varphi}^{{\prime}}$ with *p*-values < 0.01) and clustered. This procedure resulted in 11 clusters (Fig. S9). We subsetted the dataset to only include clusters 2 and 3. We identified the M1 and M2 macrophages using *SOD2* and *RGCC*, respectively (Fig. S9J, K).

For the keratinocyte dataset, we subsetted the dataset to only contain data from a single patient (labeled ADSWT11), and subsequently removed all genes expressed in less than 3 cells and removed all cells expressing less than 400 genes. We then selected features using BigSur (only selecting the top 10% of significant ${\varphi}^{{\prime}}$) and clustered, with resolution = 0.1. This procedure identified 7 clusters (Fig. S9L). We identified the keratinocyte cluster, cluster 0, using the mean expression of KR14 in the cluster (mean expression of *KRT14* > 3; Fig. S9M). We selected the keratinocyte cluster, removed all genes expressed in less than 3 cells and removed all cells expressing less than 400 genes, selected features using BigSur (using the top 5% of $\:{\varphi\:}^{{\prime\:}}$ with *p*-values < 0.01), and clustered. This resulted in 15 clusters (Fig. S9N). We subsetted the dataset to only include clusters 1, 2, 3 and 5. We identified the basal cells using *KRT5* and the granular cells using *IVL* (Fig. S9O, P).

### Subsetting of the Tabula Sapiens datasets to generate semi-synthetic datasets

To generate semi-synthetic datasets from the Tabula Sapiens datasets, we first subsetted each dataset to only contain two groups of cells. As in the previous section, we used the Leiden algorithm to cluster, with resolution = 1 if not otherwise specified.

Specifically, we downloaded the liver, pancreas, eye and lymph node datasets from the Tabula Sapiens (TS) atlas (see data availability). Since the datasets include reads from 10x Genomics and SmartSeq2, we only used the 10x Genomics reads. For each dataset, we selected the genes with the top 10% of significant $\:{\varphi\:}_{j}^{{\prime\:}}$ as features to use for dimensionality reduction.

In each dataset, we selected and clustered a cell type, displayed in Figure S13. Specifically, for the liver dataset, we removed all cells besides the hepatocytes, identified using *PCK1* expression, from patient TSP14 (clusters 0, 3, 4, 12 and 14); for the pancreas dataset, we removed all cells besides a cluster of acinar cells, identified using the expression of *CLPS*, cluster 0; for the eye dataset, we removed all cells besides clusters 1, 5 and 11, which we identified as Müller glial cells using *GLUL*; and for the lymph node dataset, we removed all cells besides cluster 19, which we identified as macrophages from their expression of *CD68*.

Subsequently, for each of these smaller subsets, we selected features (by selecting genes with the top 10% of significant $\:{\varphi\:}_{j}^{{\prime\:}}$), clustered and removed all cells besides two groups of cells. Specifically, we chose clusters 0 and 4 from the hepatocytes; clusters 0 and 1 from the acinar cells; clusters 2 and 4 from the glial cells; and clusters 0 and 1 from the macrophages (see Figs. S13 and S14A–D).

### Subsetting of datasets using HVGs to produce two clusters for semi-synthetic dataset generation

Since we generated semi-synthetic datasets using BigSur to select features, which could introduce bias, we also generated semi-synthetic datasets of similar size using HVGs. As above, we used the Leiden algorithm to cluster, with resolution = 1 if not otherwise specified.

Specifically, for each of eight datasets (the macrophage dataset, the 1M PBMC dataset, the 10k PBMC dataset, the skin dataset, and the liver, pancreas, retina, and lymph node datasets from the Tabula Sapiens atlas), we removed cells with less than 400 expressed genes and genes expressed in less than 3 cells. In addition, for the macrophage dataset, we also removed data generated from macrophages in the first stage of differentiation and data generated from the progenitor cells (labeled BM0 and AHL60 in the metadata, respectively), along with the repolarized macrophages (labeled XM2M2 and ZM2M1). For the 1M PBMC dataset, due to the size of this dataset, we also removed all cells not expressing *CD3E*. For the skin dataset, we removed all cells not sequenced from patient ADSWT11.

We then selected features using HVGs, clustered, and identified a specific cell type using known markers. Specifically, we identified T cells in the 10k PBMC dataset using *CD3E*, keratinocytes in the skin dataset using *KRT14*, hepatocytes in the liver dataset using *PCK1*, acinar cells in the pancreas using *CLPS*, Müller glial cells in the retina dataset using *GLUL* and macrophages in the lymph node dataset using *CD68* (see Fig. S18).

Then, for each clustered cell type, we selected cells from particular clusters and removed the rest of the cells. Specifically, we selected clusters 1, 3, 4, 7, 8, 9 and 14 from the PBMC dataset, clusters 4 and 10 from the T cells identified in the 1M PBMC dataset, clusters 0 and 1 from the macrophages dataset, clusters 0, 3, 5, 6, 7, 9, 10, 12, 13, 15 and 17 from patient ADSWT11, clusters 0, 1, 3, 11 and 27 from the liver dataset, clusters 1, 2, 3, 5, 6, 14 and 18 from the pancreas dataset, clusters 28 and 30 from the retina dataset, and cluster 15 from the macrophages identified in the lymph node dataset (see Fig. S18).

Finally, for each resulting subset, we selected features using HVGs. For the 10k T cell, 1M T cell, keratinocyte, and hepatocyte subsets, clusters were assigned, and the two largest clusters (0 and 1) were used to generate semi-synthetic datasets. To avoid selecting clusters with large differences in sequencing depth (see Fig. S18), for the acinar subset, clusters 1 and 4 were used to generate semi-synthetic data, and for the macrophage subset, clusters 1 and 3 were used to generate semi-synthetic data.

### Enrichment score

To calculate the enrichment score of a gene in a clustered dataset, we divide a chosen gene’s mean expression in cells of the cluster by the mean expression of the gene across all cells. In Figs. 5 and 6, we show the highest enrichment score of the dataset.

### Purity score

For a dataset in which there is *a priori* knowledge of which cells have a particular identity, one may define a purity score that evaluates the ability of unsupervised clustering to place those “target cells” together within a single cluster. After clustering, we identify the cluster that has the greatest number of target cells and then define the purity score as the fraction of target cells in that cluster.

We also calculated the probability of seeing a given purity score if the target cells were distributed in each cluster at random (the *p*-value of the purity score), using the hypergeometric distribution, which represents the probability of seeing $k$ successes with $\:n$ draws, without replacement:

$$p\left(k,M,n,N\right)=\frac{\left(\genfrac{}{}{0pt}{}{n}{k}\right)\left(\genfrac{}{}{0pt}{}{M-n}{N-k}\right)}{\left(\genfrac{}{}{0pt}{}{M}{N}\right)}$$

Where $M$ is the size of the dataset, $\:n$ is the total number of target cells, $N$ is the size of the cluster that has the greatest number of target cells and $k$ is the number of target cells in that cluster.

When displaying the purity score for simulated data (Fig. 3B and F), we present the mean of the purity scores calculated for each of the two cell types.

### Normalized entropy

The normalized entropy, a common measure of diversity, is the Shannon entropy divided by the maximum possible entropy [42]:

$$\eta\left(x\right)=-\sum_{i=1}^{n}\frac{p\left({x}_{i}\right)\text{l}\text{n}\left(p\left({x}_{i}\right)\right)}{\text{l}\text{n}\left(n\right)}$$

Where $n$ is the total number of clusters and $p\left({x}_{i}\right)$ is the probability of seeing a target cell $x$ in cluster $\:i$, or equivalently the fraction of all target cells that is in cluster $\:i$.

### Speed and memory comparisons

To compare the speed and memory usage of HVGs, SCT, FvF and BigSur when varying numbers of cells, we used the 1M PBMC dataset from Parse Biosciences. The dataset was filtered to retain only cells with more than 400 genes and genes expressed in at least 3 cells. For the comparison between HVGs and BigSur, 10 datasets with increasing numbers of cells (100, 500, 10^3^, 5 $\times$10^3^, 10^4^, 5$\:\times\:$10^4^, 10^5^ and 2.5$\:\times\:$10^5^ cells) were randomly sampled from the 1M PBMC dataset. Each sampled dataset was filtered to retain only genes expressed in at least 1 cell.

Since SCT and FvF were first implemented in R, we randomly sampled a 2.5$\:\times\:$10^5^ dataset from the filtered dataset and exported to R. Ten datasets were randomly sampled from the filtered dataset per number of cells, and the features were calculated from the sampled datasets.

To compare the speed and memory usage of the different feature selection methods when varying numbers of genes, we randomly selected 10,000 cells from the 1 M PBMC dataset and removed all genes that were not expressed in at least one cell. From this dataset, ten datasets with increasing numbers of genes (100, 500, 10^3^, 5$\:\times\:$10^3^, 10^4^, 2$\:\times\:$10^4^, and the maximum number of genes in this dataset, 21,819) were randomly sampled. We exported the initial 10,000 cell dataset to R and generated ten datasets with the same numbers of genes as in python.

The total memory used by BigSur and HVGs was recorded by the tracemalloc package which is included in python. The total memory used by FvF and SCT was recorded by the profmem package [48].

All real and semi-synthetic dataset results were generated on an iMac Pro (2017) with 10 3 Ghz Intel Xeon W cores and 256 GB of RAM running Ventura 13.2.1.

## Supplementary Information

Below is the link to the electronic supplementary material.

Supplementary Material 1

## Data Availability

No new data were generated as part of this study. The 10k PBMC dataset was downloaded from 10x Genomics, https://cf.10xgenomics.com/samples/cell-exp/6.1.0/10k_PBMC_3p_nextgem_Chromium_X/10k_PBMC_3p_nextgem_Chromium_X_raw_feature_bc_matrix.h5. The 1 million PBMC dataset was downloaded from Parse Biosciences, https://support.parsebiosciences.com/hc/en-us/articles/7704577188500-How-to-analyze-a-1-million-cell-data-set-using-Scanpy-and-Harmony. Retina [32], macrophage [49] and keratinocyte [40] datasets were downloaded from GEO (GSE137537, GSE164498 and GSE141526 respectively). Additional datasets were downloaded from the Tabula Sapiens Human Cell Atlas [41]. The metadata for each dataset, including the cell barcodes of each semi-synthetic dataset, are included in supplementary data. Code is freely available as a python package on GitHub: https://github.com/landerlabcode/BigSur. An R version can be found at: https://github.com/landerlabcode/BigSurR. A Mathematica version can be found at: https://github.com/landerlabcode/BigSurM. A tutorial on how to use BigSur is included as part of the python package.

## Abbreviations

scRNAseq: Single cell transcriptomics
BigSur: Basic informatics and gene statistics from unnormalized reads
PCs: Principal components
PCA: Principal component analysis
UMAP: Uniform manifold approximation and projection
SVM: Support vector machine
ARI: Adjusted Rand index
NMI: Normalized mutual information
PBMC: Peripheral blood mononuclear cell
HVGs: Highly variable genes
Treg: T regulatory cell
TS: Tabula Sapiens
UMI: Unique molecular identifier
FDR: False discovery rate
FvF: FindVariableFeatures
SCT: SCTransform
