# Supplementary material for: Statistically principled feature selection for single cell transcriptomics
Source: BMC Bioinformatics. 2025 Oct 2;26:238. doi: 10.1186/s12859-025-06240-y (PMC12490061; doi:10.1186/s12859-025-06240-y)
Supplement: Supplementary file 1 — Supplementary Material 1 [file 12859_2025_6240_MOESM1_ESM.pdf]

Supplementary Figures

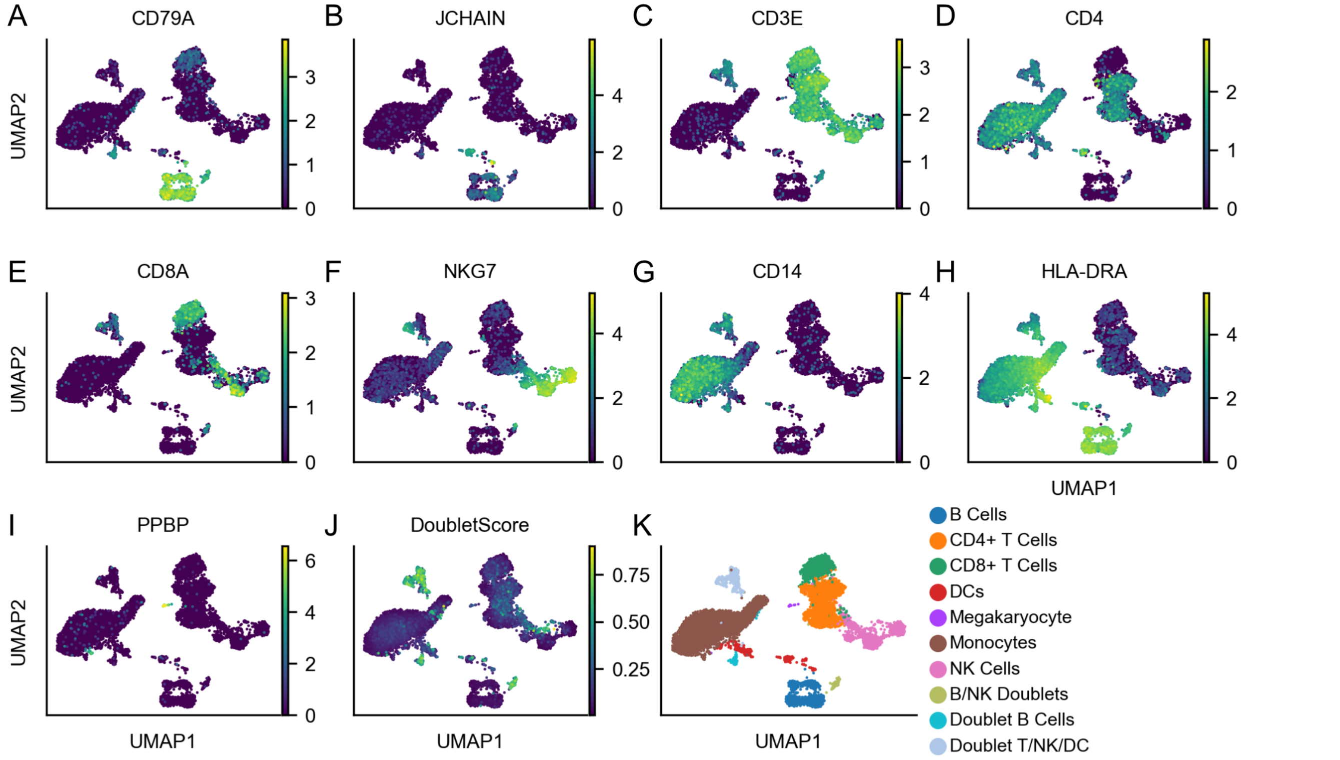

Supplementary Figure 1: Celltype identification in the PBMC dataset. UMAPs of the 10k PBMC dataset, with cells colored by expression of different marker genes, along with the doublet score calculated by scrublet and the cell type identities. Features were selected using HVGs defaults. **A – B.** *CD79A* and *JCHAIN* were used to identify B cells. **C – E.** Markers to identify the T cells: *CD3E* for all T cells, *CD4* for CD4+ T cells, *CD8A* for CD8+ T cells. **F**. *NKG7* was used to identify NK cells. **G.** *CD14* was used to identify monocytes. **H.** Co-expression of *HLA-DRA* and *CD14* was used to identify dendritic cells (DCs). **I.** *PPBP* was used to identify Megakaryoctes. **J.** Scrublet was used to calculate the doublet score for each cell (see methods). **K.** Celltypes identified using the above markers; same panel as in Figure 1A.

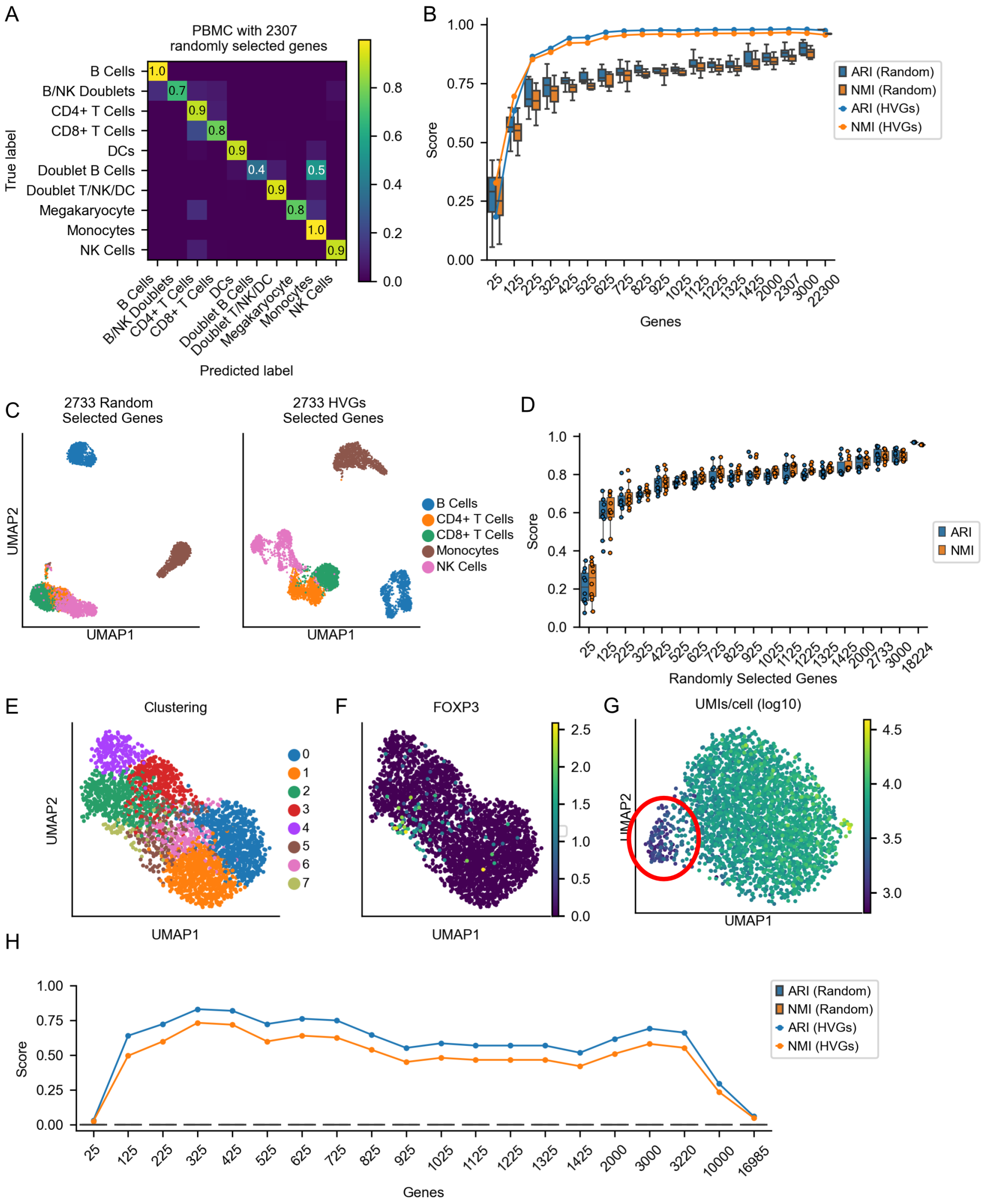

Supplementary Figure 2: Cell types can be identified using randomly selected features in some datasets. A. The confusion matrix of predictions made by a linear support vector machine (SVM) trained on the 50 top principal components (PCs) of the PBMC dataset from 2307 randomly selected genes. The rows of the confusion matrix sum to 1. The fraction of correct predictions (diagonal) are displayed in each tile, along with the fraction of doublet B cells predicted as monocytes. **B.** Adjusted Rand index (ARI) and normalized mutual information (NMI) scores of SVMs trained on the 50 top PCs of the PBMC dataset for varying number of genes ranked by HVGs. The ARI and NMI scores from randomly selected genes (data from Figure 1B) are included for comparison. **C. – D.** The cell types numbering less than 1000 in the PBMC dataset were removed. The remaining cell types were randomly downsampled to 1000. Panel C displays the UMAPs of this dataset, calculating using either randomly selected genes (left) or HVGs (right). Panel D shows the ARI and NMI scores of SVMs trained on PCs calculated from increasing numbers of randomly selected genes. **E.** UMAP of CD4+ T cells from the PBMC dataset, generated by selecting features using HVGs and clustering using Leiden. **F.** Feature plot of *FOXP3* expression of the CD4+ T cells. **G.** UMAP of CD4+ T cells, calculated by randomly selecting 350 genes and calculating their PCs (same as in Figure 1C). A small subpopulation of lowly sequenced cells (circled in red) separated out from the other cells. **H.** Adjusted Rand index (ARI) and normalized mutual information (NMI) scores of SVMs trained on the 50 top PCs of the CD4+ T cell dataset for varying number of genes ranked by HVGs. The ARI and NMI scores from randomly selected genes (data from Figure 1D) are included for comparison; the default number of features selected by HVGs was 3220.

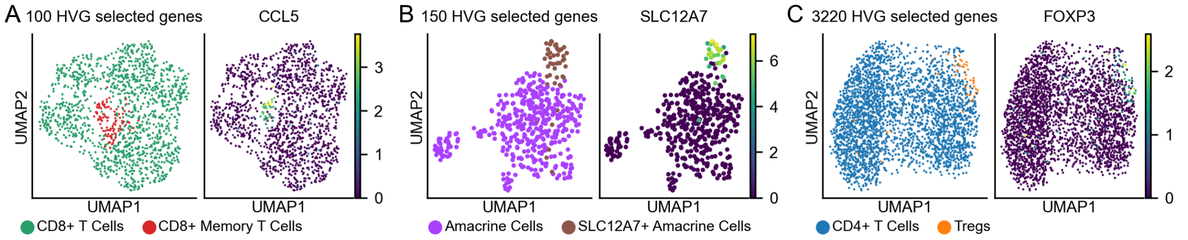

Supplementary Figure 3: Identification of clusters used in Figure 2. A. UMAPs of CD8+ T cells from the PBMC dataset, generated by selecting the top 100 features ranked by HVGs and clustering using the Leiden algorithm. The CD8+ memory T cell population was identified by expression of *CCL5*. **B.** UMAPs of amacrine cells, which were subset from the retina dataset. The top 150 genes ranked by HVGs were selected and the cells were clustered using the Leiden algorithm. A cluster of *SLC12A7*-expressing amacrine cells was identified. **C.** UMAPs of CD4+ T cells from the 10k PBMC dataset. The top 3220 HVG features were selected and the cells were clustered using the Leiden algorithm. Tregs were identified using *FOXP3* expression.

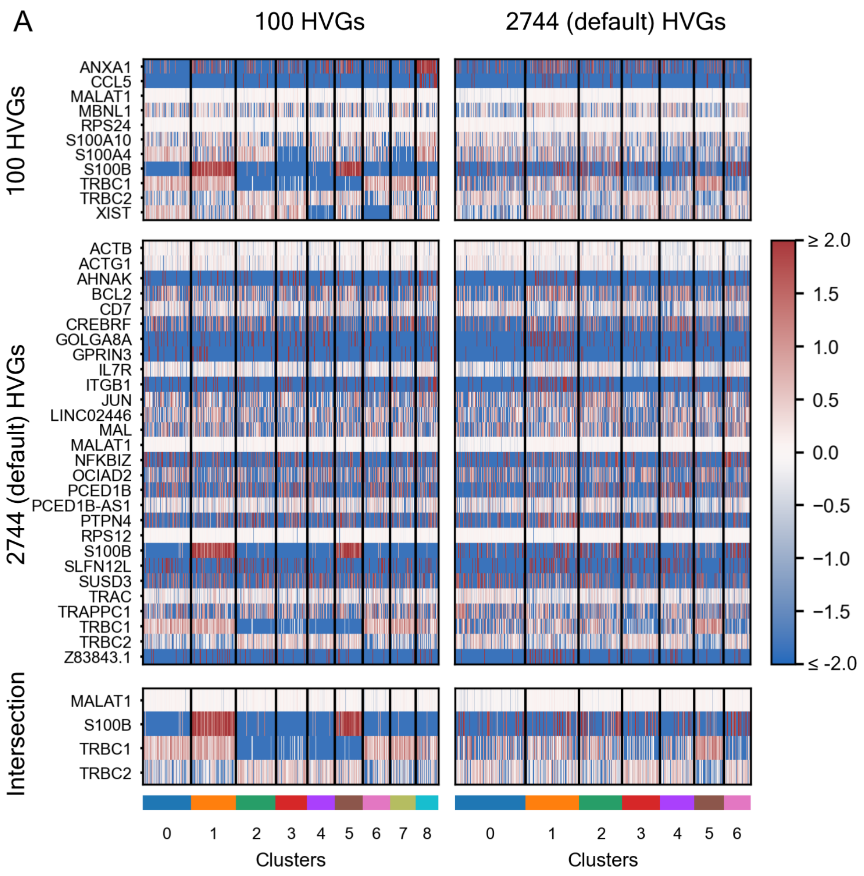

Supplementary Figure 4: Changes in differentially expressed genes in the CD8+ T cell dataset with varying number of selected features. Log_2 fold change of differentially expressed genes (DEGs) of the normalized expression in each cell to the mean normalized expression in the CD8+ T cells, for different sets of clusters. The CD8+ T cells were clustered by the Leiden algorithm using either the top 100 HVGs or the default number of HVGs. The differentially expressed genes (DEGs) for each cluster were identified using an FDR-adjusted Mann-Whitney-U *p*-value threshold of 0.05. If more than five genes had a *p*-value below 0.05, only the top five DEGs (ranked by average log foldchanges) are shown. For genes in which there was no expression in a cell, the log2 fold change was set to -2.

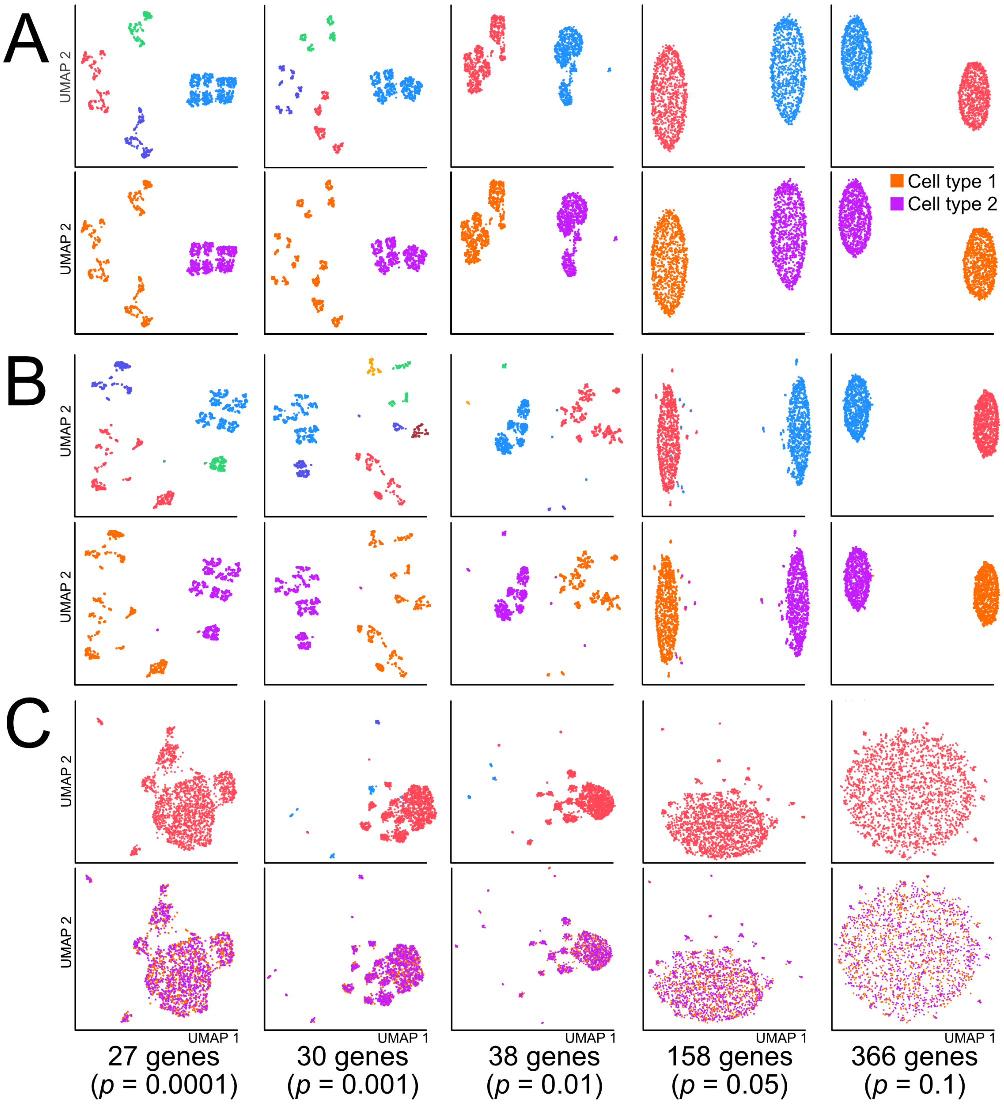

Supplementary Figure 5: Clustering of simulated data using different feature selection methods and cutoffs. UMAPs of simulated data with 100 truly variable genes (dataset used in Fig. 3B-F) calculated using different feature selection methods (BigSur, HVGs and randomly selected genes in panels A, B and C respectively) and number of genes (columns). For each panel, the top row displays cells colored by cluster assignment (using a Leiden resolution of 0.1), and the bottom row displays cells colored by cell type identity. For each UMAP, the counts matrix was log-normalized (see "Dimensionality reduction and clustering" section of the methods), features were selected, the top 50 PCs were selected (or all PCs if the number of selected features is lower than 50) and used to calculate the UMAP coordinates. **A**. UMAPs were calculated using varying numbers of genes using *p*-value cutoffs calculated using BigSur. **B – C**. UMAPs calculated using the same number of features as in panel A. Features were selected by HVGs (panel B) or at random (panel C).

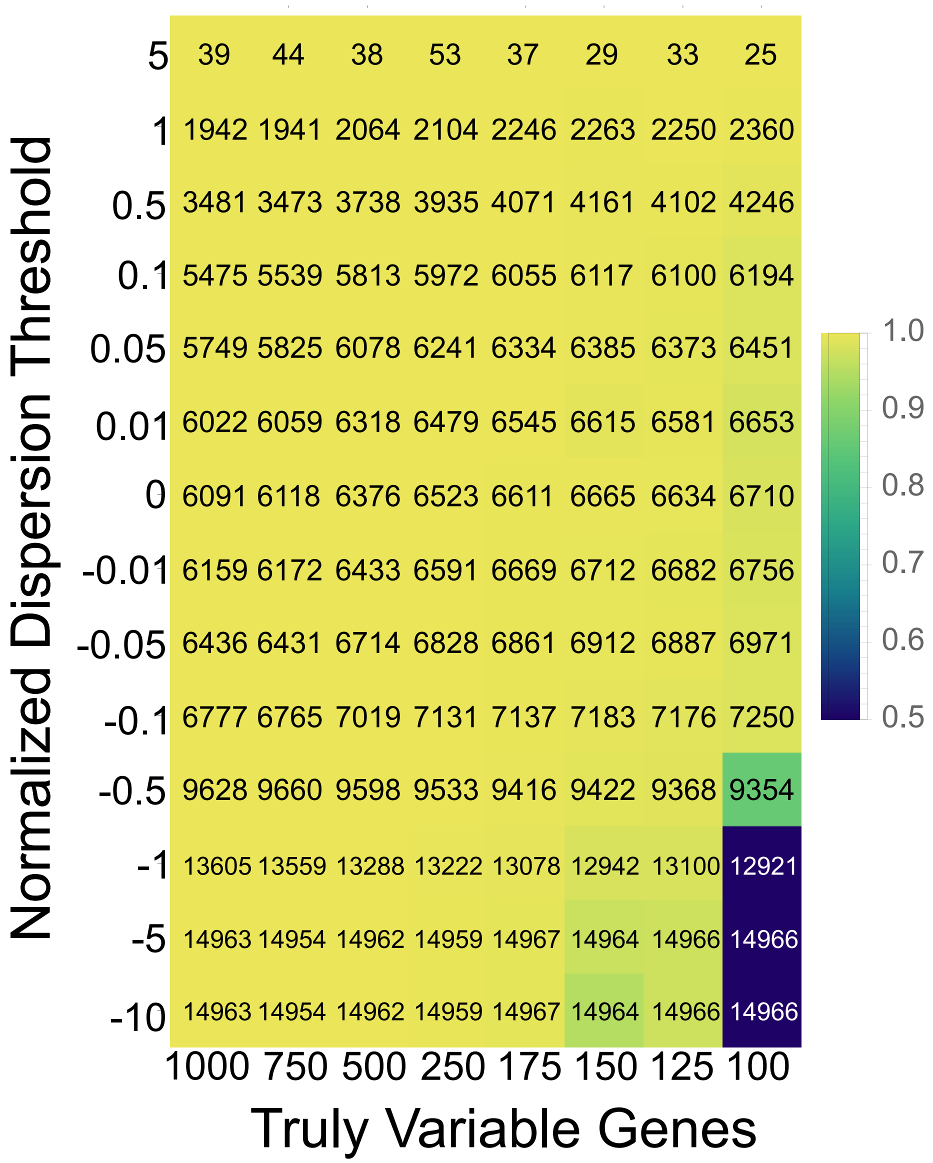

Supplementary Figure 6: Purity scores of simulated data when using HVGs. Heatmap of purity scores of the simulated data in Figure 3B yielded by features selected by HVGs at different normalized dispersion thresholds. Tile color indicates purity score. Numbers overlaid on each tile indicate the number of features selected. Note that HVGs selects all genes with normalized dispersion > 0.5 by default.

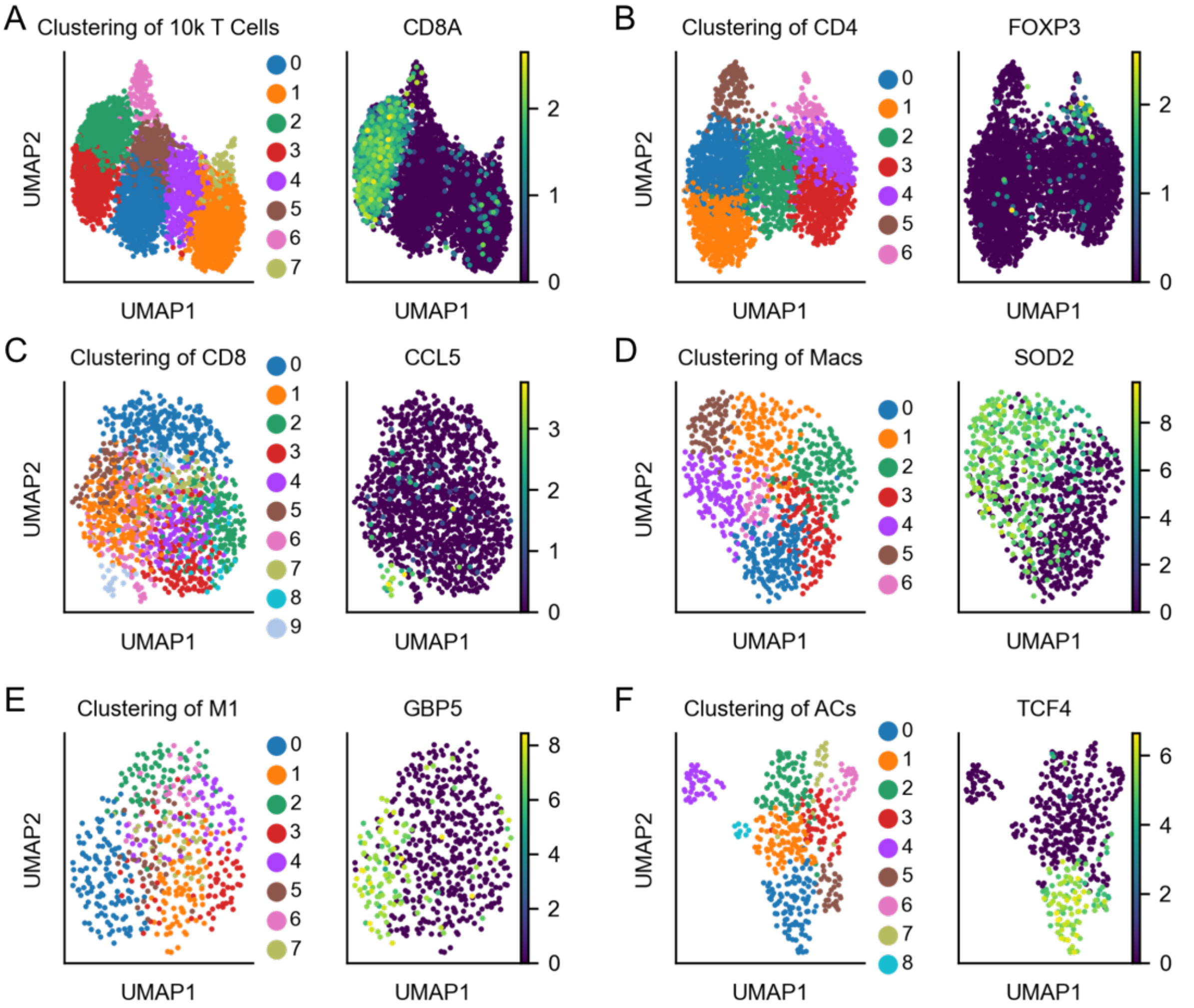

Supplementary Figure 7: Markers and clusters used for enrichment calculations.
UMAPs of datasets used for the enrichment calculations in Figure 4J and K are shown, along with the marker used to calculate enrichment. Clusters were assigned using the Leiden algorithm with resolution = 1. For the 10k T cell, CD4 T cell, Macs (macrophages), and AC datasets, the genes with top 10% of ϕ' (0.9 quantile cutoff) and with p-values < 0.05 were used to calculate principal components (PCs). For the CD8 T cell and M1 macrophage datasets, the genes with top 1% of ϕ' (0.99 quantile cutoff) were used to calculate PCs.

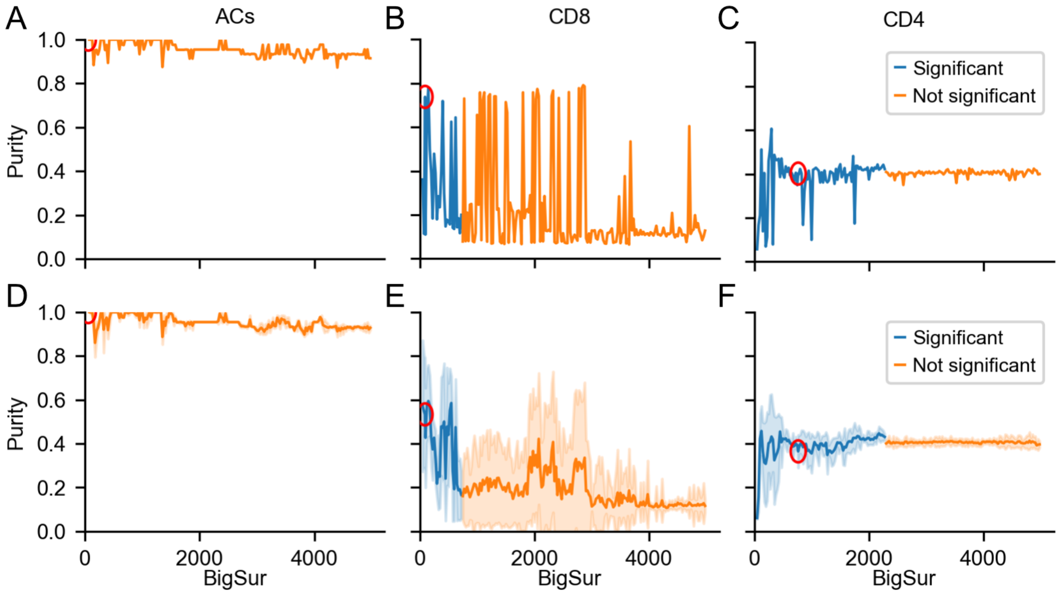

Supplementary Figure 8: Purity of Figure 2 datasets using BigSur. The purities of datasets shown in Figure 2 with varying number of genes selected by BigSur are shown. The genes were first ranked by significance of ϕ', then by magnitude of ϕ'. For each set of features, clusters were assigned using the Leiden algorithm and the purity score was calculated. The red circles mark the default selection of genes for each dataset. **A – C.** Purity of the ACs (panel A), CD8 (panel B) and CD4 (panel C) datasets, using the default Leiden seed of 0. **D – F.** Mean and standard deviation of the purity of the ACs (panel D), CD8 (panel E) and CD4 (panel F) datasets using 50 randomly selected starting seeds for the Leiden algorithm.

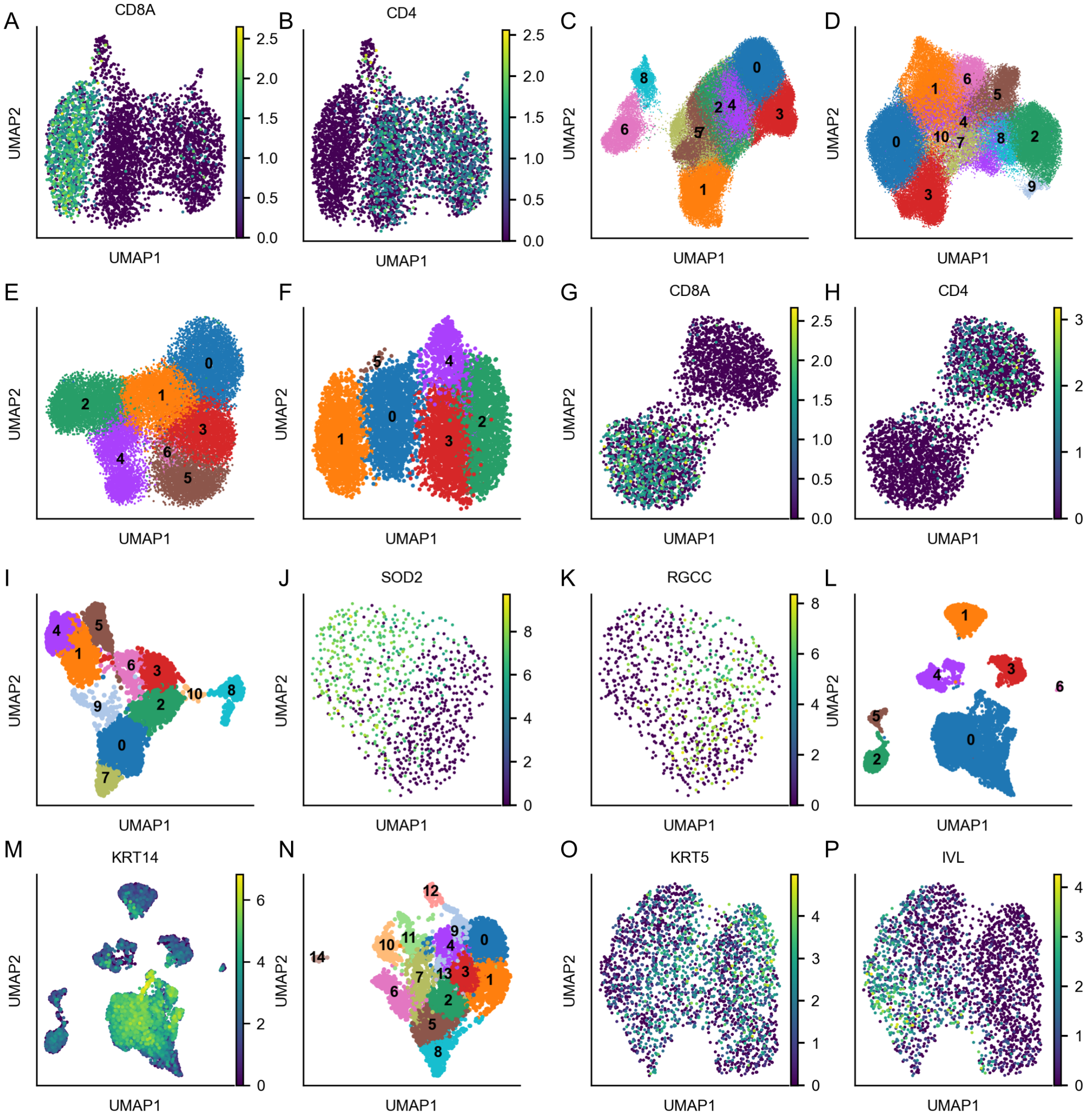

Supplementary Figure 9: Intermediate steps to downsample datasets used for semi-synthetic dataset generation. The intermediate steps to downsample datasets subsequently used to generate semi-synthetic data are visualized, along with the marker genes used to identify the two groups of cells. The specific procedure downsampling procedure is detailed in the methods. **A – B.** UMAPs of the T cells from the 10k PBMC dataset (calculated from the top 10% of significant ϕ'), colored by expression of *CD8A* and *CD4*. **C – F.** UMAPs of the four successive downsampling steps for the 1M T cell dataset. Feature were selected using BigSur, with various cutoffs (see methods). **G – H.** UMAPs of the CD8 and CD4 T cells from the downsampled 1M T cells dataset (calculated from the top 10% of significant ϕ'), identified by expression of *CD8A* (panel G) and expression of *CD4* (panel H). **I.** UMAP of the macrophage dataset (calculated from the top 10% of ϕ' with *p*-values < 0.01). **J – K.** UMAP of clusters 2 and 3 of the macrophage dataset (previously shown in panel I; UMAPs were calculated from the top 10% of significant ϕ'). The M1 macrophages were identified using *SOD2* expression (panel J) and the M2 macrophages were identified using expression of *RGCC* (panel K). **L – M.** UMAP of the cells from patient ADSWT11 (included in the skin dataset), calculated using the top 10% of significant ϕ'. Clusters shown in panel L were assigned using resolution = 0.1. The keratinocytes were identified using expression of *KRT14* (shown in panel M). **N.** UMAP of the keratinocytes (cluster 0 in panel L), calculated using the top 5% of ϕ' with *p*-values < 0.01. **O – P.** UMAP of clusters 1, 2, 3 and 5 from the keratinocytes (shown in panel N), calculated from the 10% of significant ϕ'. Basal cells were identified using *KRT5* expression (panel O) and granular cells were identified using *IVL* (panel P).

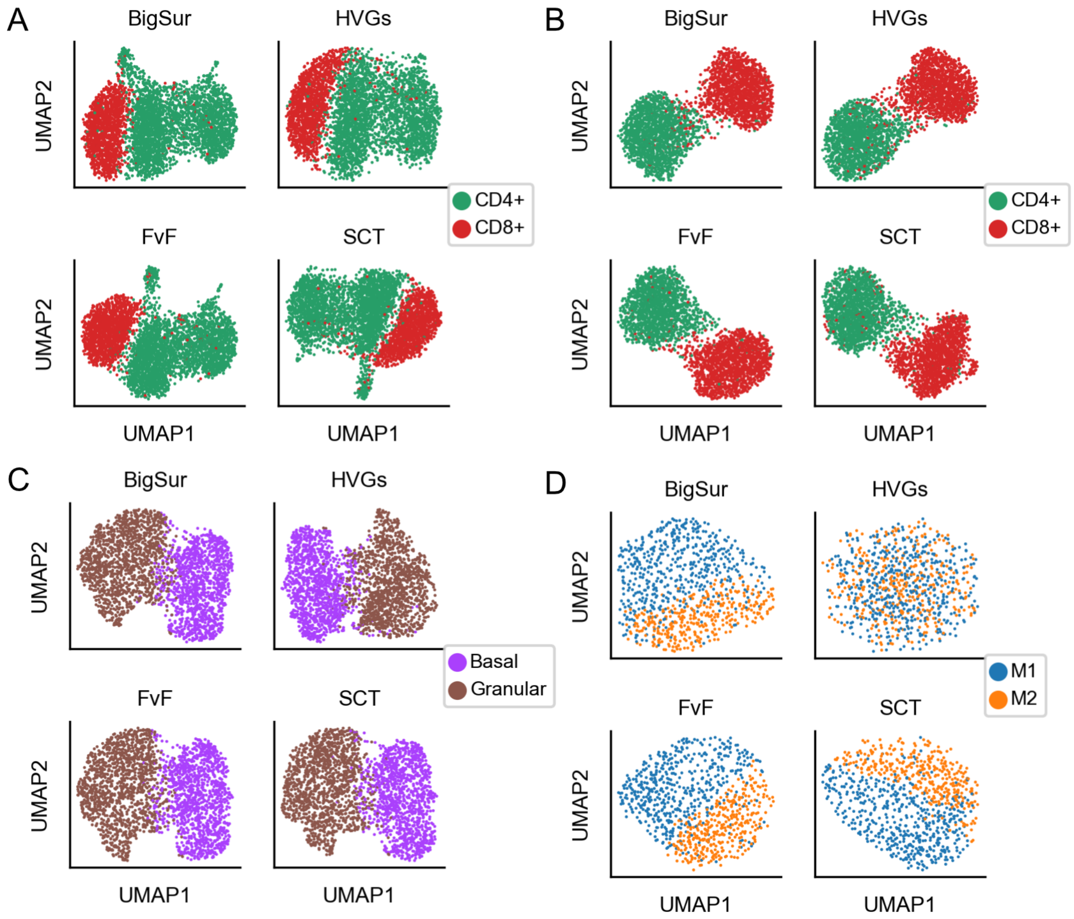

Supplementary Figure 10: Feature selection of four methods on the four datasets used to generate semi-synthetic datasets discussed in Figure 6A-H. UMAPs of the dowsampled 10k T cells (panel A), 1M T cells (panel B), keratinocyte (panel C) and macrophage (panel D) datasets (intermediate steps are shown in Figure S8 and detailed in methods). For each dataset, features were selected using either BigSur (selecting the top 10% of significant ϕ'), HVGs, SCT or FvF.

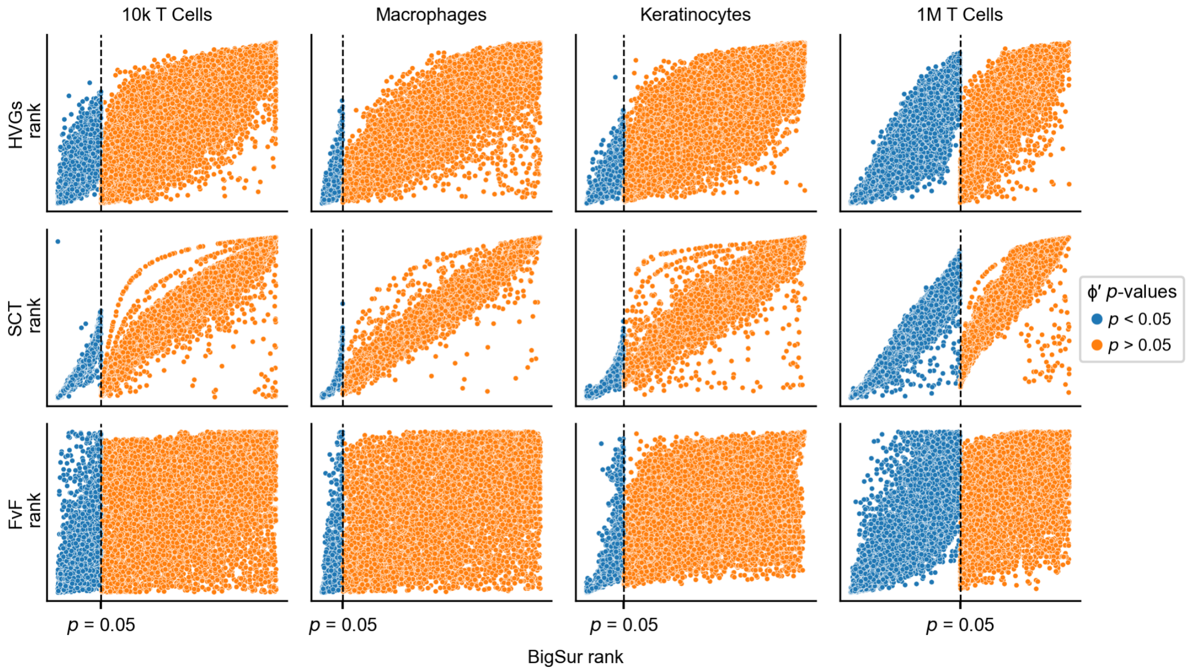

Supplementary Figure 11: Ranking of features for the four datasets used to generate semi-synthetic datasets discussed in Figure 6A-H. Ranking of features by FvF, SCT and HVGs plotted against the ranking of features by BigSur, for each dataset shown in Figure S9. To rank the features using BigSur, genes were first binned by ϕ' p-value, then ranked in order of decreasing ϕ'.

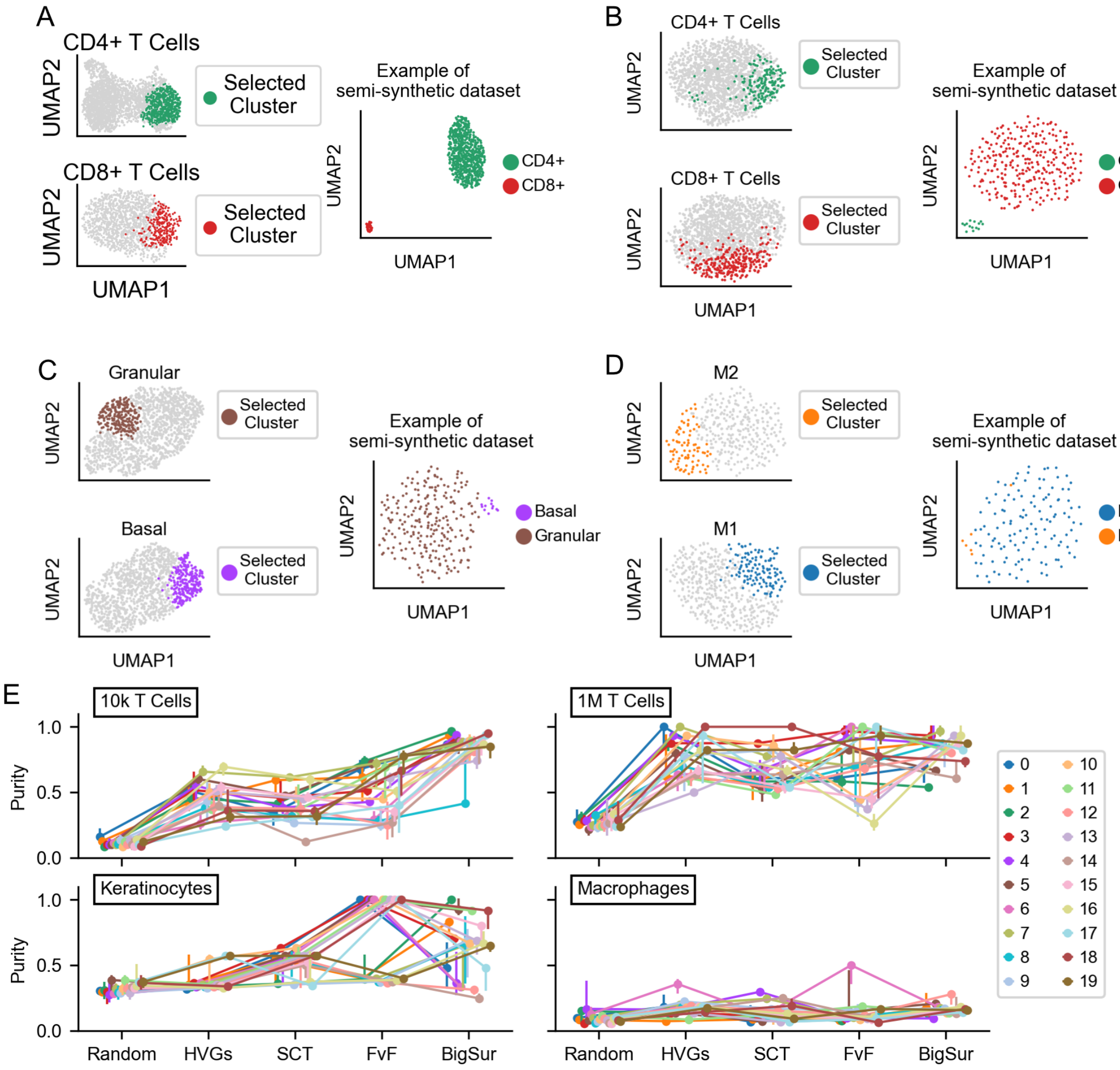

Supplementary Figure 12. Visualization of semi-synthetic dataset generation and their purity scores. A – D. UMAPs of intermediate steps (described in methods) for semi-synthetic data generation, along with an example of a semi-synthetic dataset, for the 10k T cell (panel A), 1M T cell (panel B), keratinocyte (panel C), and macrophage (panel D) datasets. Each UMAP was calculated from the top 10% of significant ϕ'. **E.** For each semi-synthetic dataset, features were selected using four different methods using their defaults (see main text for BigSur's cutoffs), clustered using the Leiden algorithm with 40 different starting seeds, and calculated the purity score of the rare cells. The first 10 datasets (0-9) are the datasets shown in Figure 6D – H. As in Figure 6, the dots represent medians, and the bars represent interquartile range.

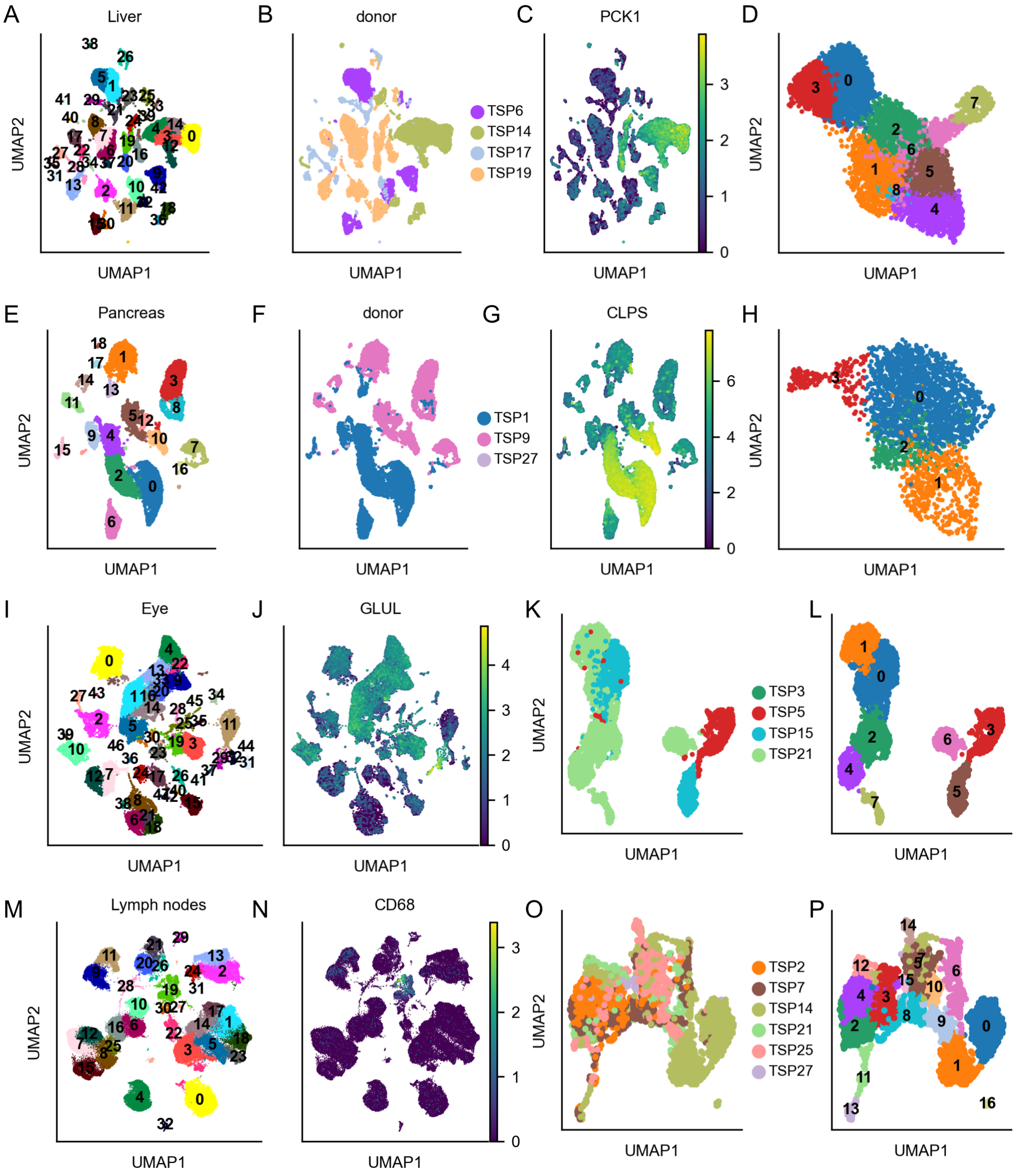

Supplementary Figure 13: Downsampling steps for the Tabula Sapiens datasets. UMAPs of downsampling steps of the liver, pancreas, eye and lymph node datasets from the Tabula Sapiens human cell atlas, see methods for details. For each UMAP, the top 10% of significant ϕ' were selected using BigSur and clustering was done using the Leiden algorithm (with resolution = 1 if not otherwise specified). **A – C.** UMAPs of the liver dataset. Cells are either colored by cluster assignments (panel A), donor IDs (panel B) or gene expression of *PCK1* (panel C). **D.** UMAP of clusters 0, 3, 4, 12 and 14 from the liver dataset. **E – G.** UMAP of the pancreas dataset. Cells are either colored by cluster assignment (which was done using a resolution = 0.3; panel E), donor IDs (panel F) or gene expression of *CLPS* (panel G). **H.** UMAP of cluster 0 of the pancreas dataset. **I – J.** UMAPs of the eye dataset, with cells colored by either cluster assignment (panel I) or gene expression of *GLUL* (panel J). **K – L.** UMAP of clusters 1, 5 and 11 of the eye dataset. Cells are colored by donor IDs (panel K) or cluster labels (panel L). **M – N.** UMAP of the lymph node dataset. Cells are colored by clusters (panel M) or expression of CD68. **O – P.** UMAP of cluster 19 from the lymph node dataset. Cells are colored by donor IDs (panel O) or cluster labels (panel P).

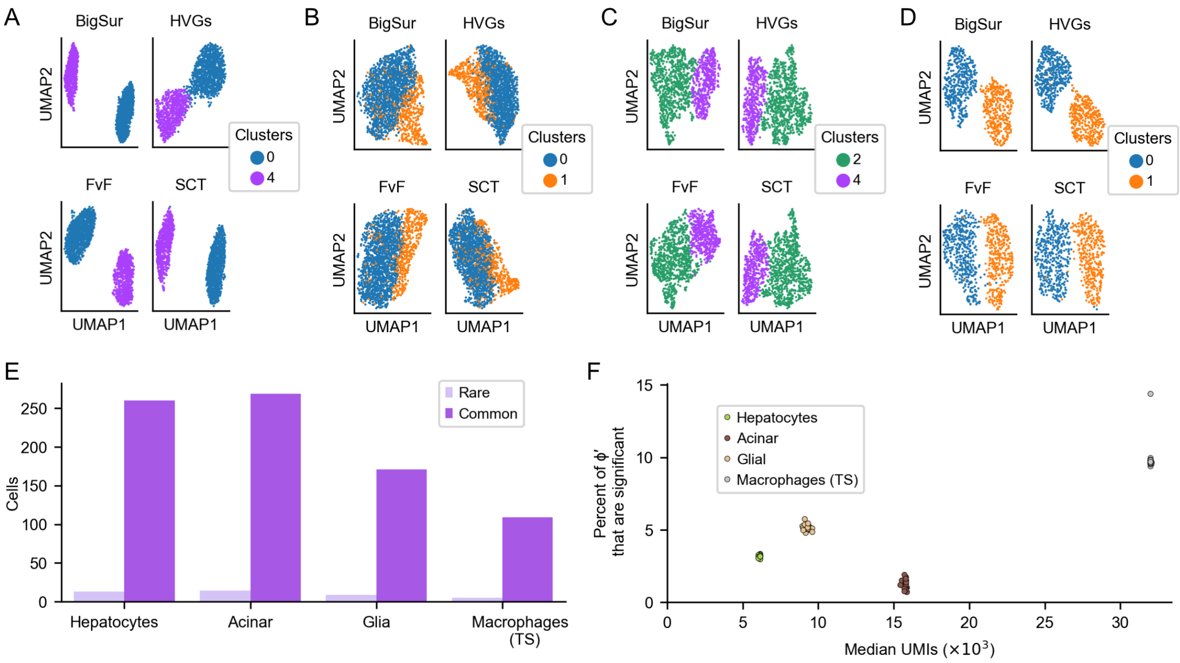

Supplementary Figure 14. Statistics and results of the Tabula Sapiens semi-synthetic datasets. The Tabula Sapiens (TS) datasets (the liver, pancreas, eye and lymph nodes datasets, see main text) were downsampled to only include two clusters of a cell type (hepatocytes, acinar cells, glia cells, and macrophages, respectively), detailed in the methods. **A – D.** UMAPs of the four downsampled datasets. For each dataset, features were selected, from which PCs and subsequently UMAP coordinates were calculated. The liver dataset clusters are shown in panel A; the pancreas dataset clusters are shown in panel B; the eye dataset clusters are shown in panel C; and the lymph node clusters are shown in panel D. **E – F.** Semi-synthetic datasets were generated from the TS datasets using the same procedure as in Figure 6 (see methods). For each semi-synthetic dataset, the number of rare and common cells for each semi-synthetic dataset is displayed in panel E, and the percent of ϕ' that are significant and median UMI/cell are displayed in panel F.

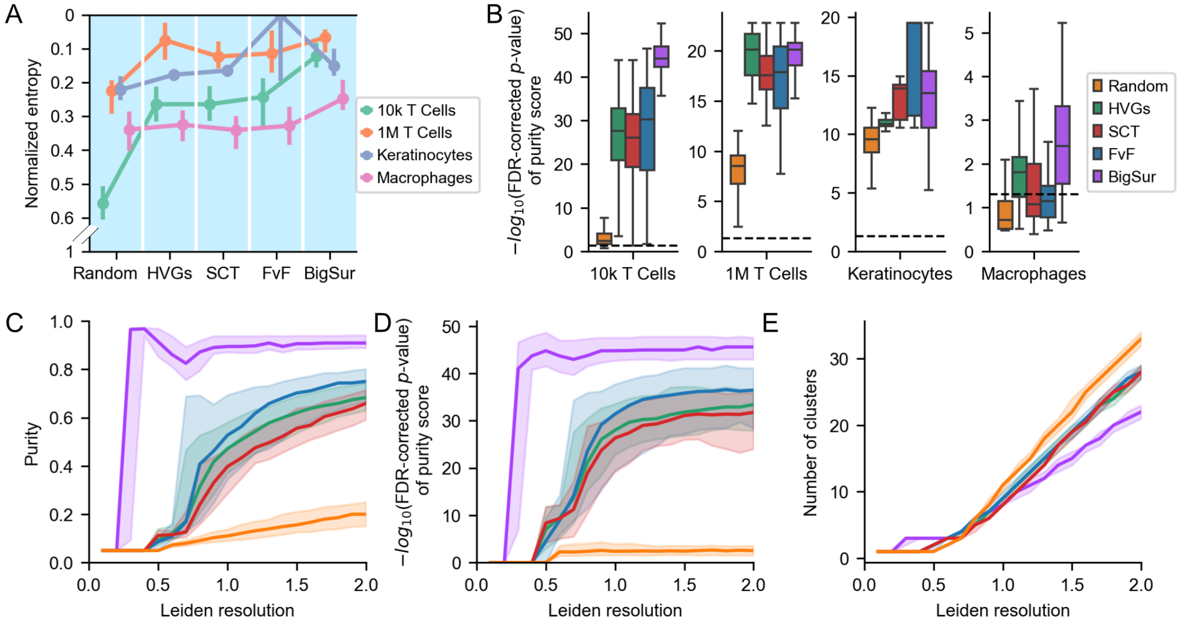

Supplementary Figure 15: Validation of purity score. For each semi-synthetic dataset whose purities are shown in Figure 6D-H, features were selected (using either HVGs, SCT, FvF or BigSur or at random) and clusters were assigned with 40 different Leiden starting seeds. **A.** The normalized entropy of the rare cells (see methods). **B.** *p*-values of the purity scores for each set of clusters (see methods). The dashed line represents $p = 0.05$. **C-E.** For each semi-synthetic generated from the 10k T cells dataset, features were selected using different methods and clustering was done using the Leiden algorithm, using 40 different starting seeds, with varying resolution. The resulting purity scores are shown in panel C, their *p*-values are shown in panel D and the number of clusters at each resolution is shown in panel E. Each plot displays the median and interquartile range (IQR) of the relevant measurement. The colors correspond to the feature selection method used, see panel B.

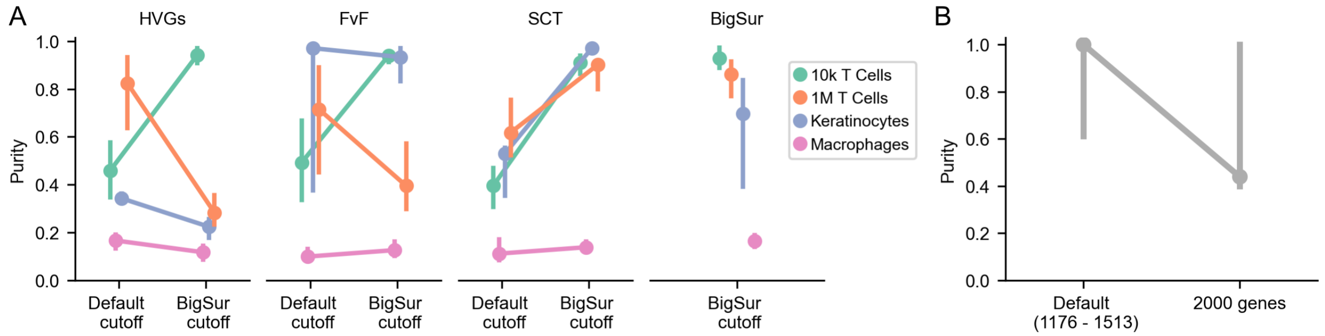

Supplementary Figure 16: Change in purity score when varying numbers of genes selected by other feature selection methods. A. Median and IQRs of purities of the semi-synthetic datasets discussed in Figure 6D-H, yielded by using each method's default cutoff (data from Figure 6H) or by limiting the number of genes selected to the number of genes BigSur selected ("BigSur cutoff"; see Figure 6C and main text). **B.** Purity scores of the macrophage (TS) semi-synthetic datasets with 10% rare to total cells yielded by using the default cutoffs chosen by BigSur (data from Figure 6J) or the 2000 genes with the highest ϕ' (without using p-value cutoff).

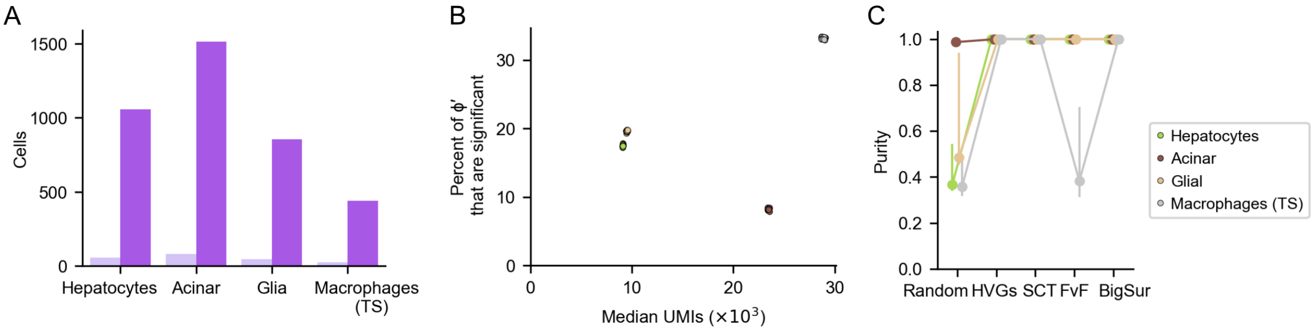

Supplementary Figure 17: Statistics and results of larger TS datasets. Large semi-synthetic datasets from the TS datasets were generated (see methods). **A-B.** The number of rare and common cells for each semi-synthetic dataset are shown in panel A, and the percent of ϕ' that are significant and median UMI/cell of these semi-synthetic datasets are shown in panel B. See panel C for legend. **C.** For each of the large semi-synthetic TS datasets, whose statistics are shown in panels H and I, features were selected, clusters were assigned using the Leiden algorithm, with 40 different Leiden starting seeds, and the purity scores were calculated, as in Figure 6. When using BigSur, the top 10% of significant ϕ' were selected.

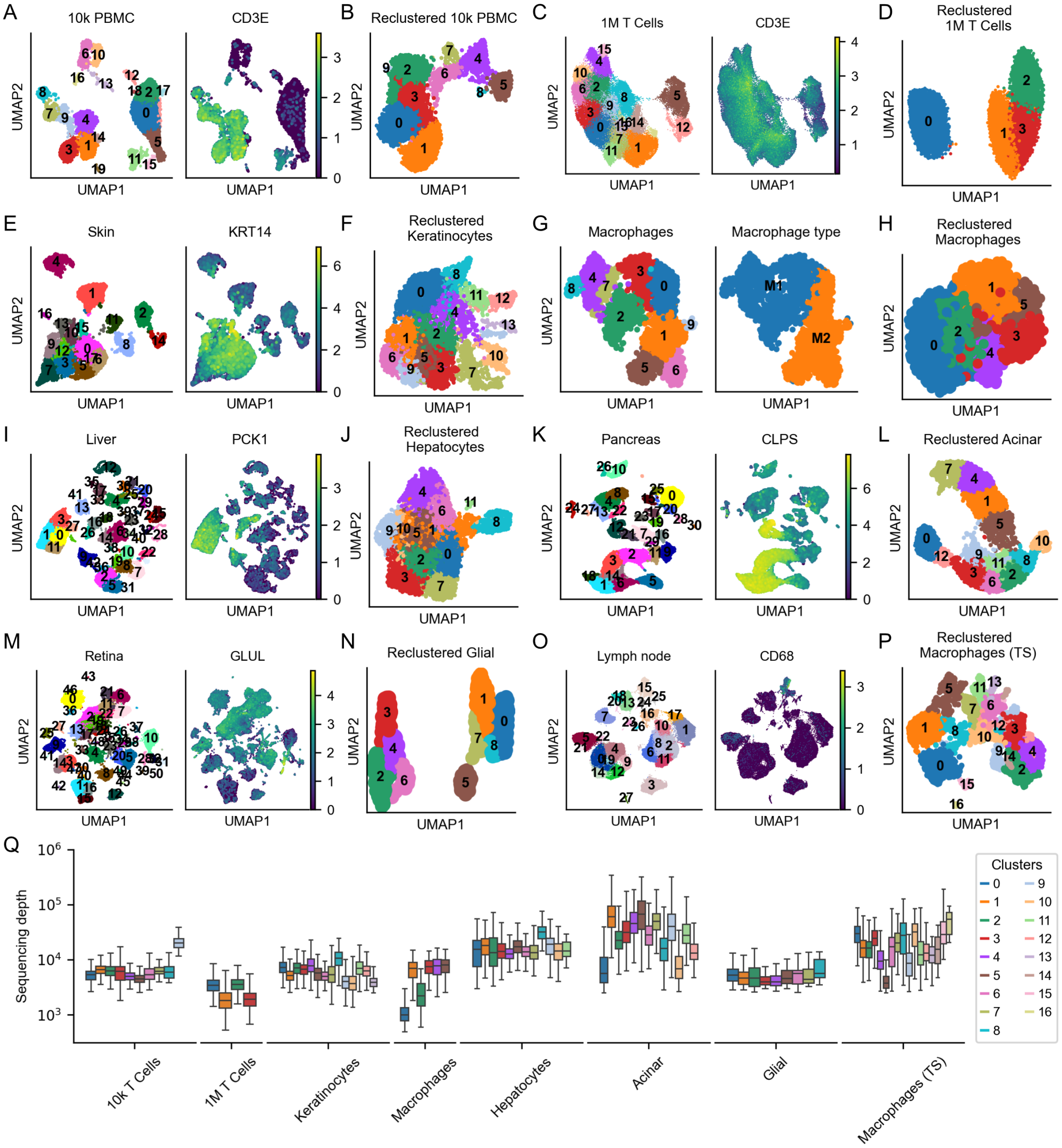

Supplementary Figure 18: Downsampling of eight datasets using HVGs. UMAPs of the eight datasets discussed in Figure 6 (the 10k T cells, 1M T cells, keratinocyte and macrophage datasets, along with the four datasets from the Tabula Sapiens atlas), along with UMAPs of subsets of cells from these datasets. For each UMAP, features were selected using HVGs (using defaults) and clustering was done using the Leiden algorithm (with resolution = 1). **A.** UMAPs of the 10k PBMC dataset, with cells colored by cluster assignment (left) or gene expression of *CD3E* (right). **B.** UMAP of cells assigned to clusters 1, 3, 4, 7, 8, 9 and 14 from the 10k PBMC dataset. **C.** UMAPs of the cells expressing *CD3E* in the 1M PBMC dataset. **D.** UMAP of cells assigned to clusters 4 and 10 displayed in panel C. **E.** UMAPs of the cells sequenced from patient ADSWT11 from the skin dataset. **F.** UMAP of cells assigned to clusters 0, 3, 5, 6, 7, 9, 10, 12, 13, 15 and 17 shown in panel E. **G.** UMAPs of macrophages from the macrophage dataset, excluding macrophages in the first stage of differentiation, progenitor cells, and repolarized macrophages (see methods for details). Cells are colored by cluster assignment (left) or macrophage polarization (right). **H.** UMAP of cells assigned to clusters 0 and 1 in the macrophage dataset. **I.** UMAPs of cells from the liver Tabula Sapiens (TS) dataset. **J.** UMAP of cells assigned to clusters 0, 1, 3, 11 and 27 in the liver dataset. **K.** UMAPs of cells from the pancreas TS dataset. **L.** UMAP of cells assigned to clusters 1, 2, 3, 5, 6, 14 and 18 in the pancreas dataset. **M.** UMAPs of cells from the retina TS dataset. **N.** UMAP of cells assigned to clusters 28 and 30 in the retina dataset. **O.** UMAPs of cells from the lymph node TS dataset. **P.** UMAP of cells assigned to cluster 15 in the lymph node TS dataset. **Q.** Sequencing depth (total UMI/cell) of all clusters in each dataset. Note the large differences in sequencing depth of clusters 0 and 1 in the macrophage and acinar datasets.

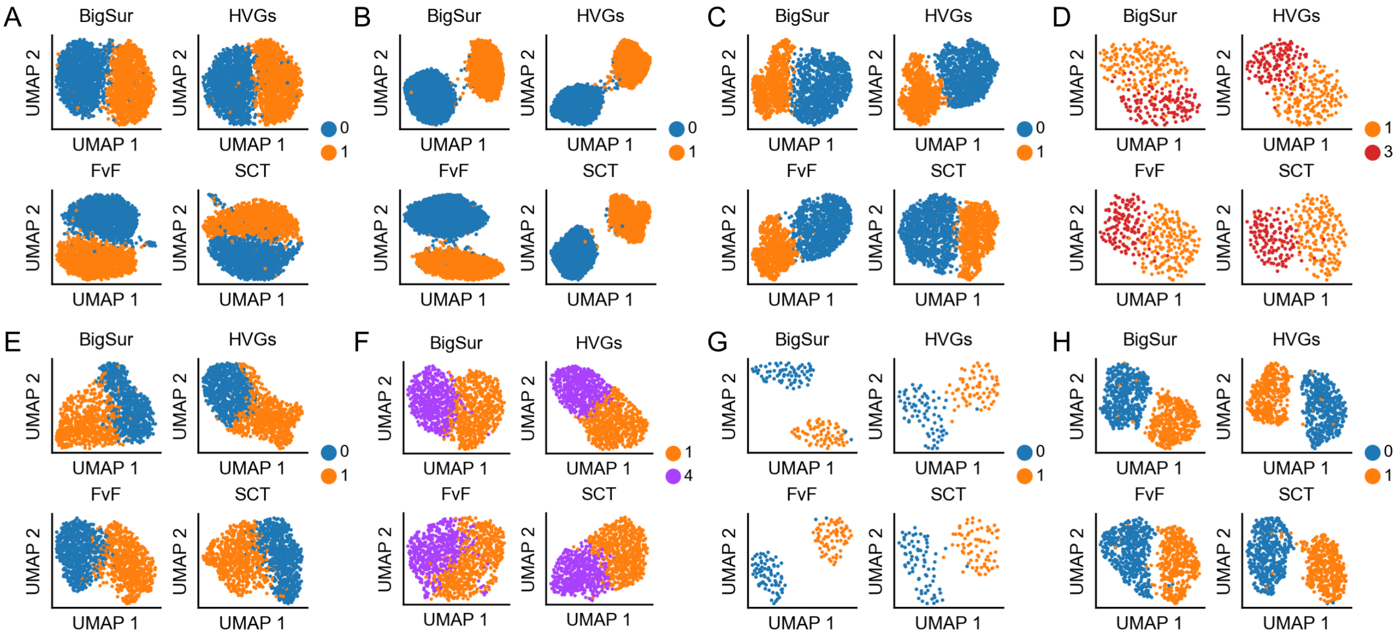

Supplementary Figure 19: Feature selection applied to eight downsampled datasets generated using HVGs. UMAPs of datasets which were used to generate semi-synthetic datasets. For each dataset, features were selected using BigSur (selecting the top 10% of significant ϕ'), HVGs, SCT or FvF. The cluster assignments for each dataset are displayed in Figure S15. **A.** UMAPs of T cells from the 10k PBMC dataset (cluster assignments shown in Figure S15B). **B.** UMAPs of T cells from the 1M PBMC dataset (cluster assignments in Figure S15D). **C.** UMAPs of keratinocytes from the skin dataset (cluster assignments in Figure S15F). **D.** UMAPs of macrophages from the macrophage dataset (cluster assignments in Figure S15H). **E.** UMAPs of hepatocytes from the liver TS dataset (cluster assignments in Figure S15J). **F.** UMAPs of acinar cells from the pancreas TS dataset (cluster assignments in Figure S15L). **G.** UMAPs of glial cells from the retina TS dataset (cluster assignments in Figure S15N). **H.** UMAPs of macrophages from the lymph node TS dataset (cluster assignments in Figure S15P).

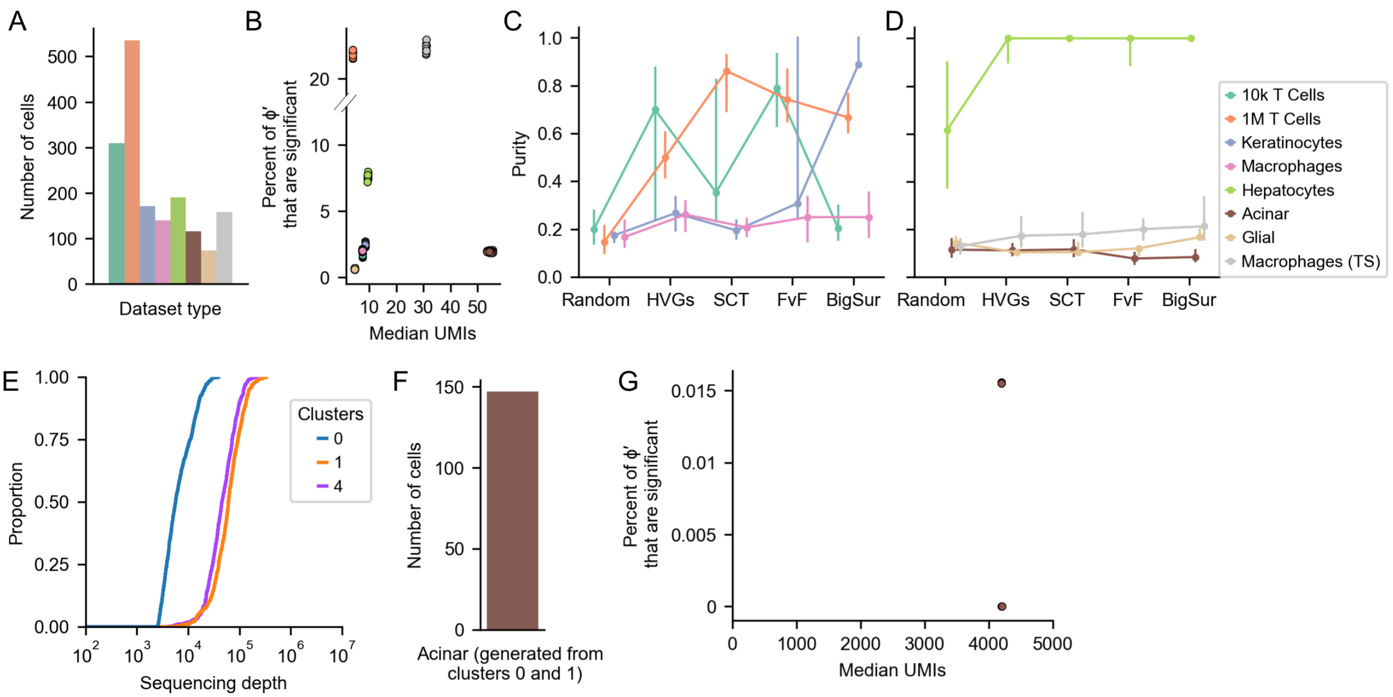

Supplementary Figure 20: Results from semi-synthetic datasets generated using HVGs. For each of eight datasets, 20 semi-synthetic datasets were generated using HVGs (the intermediate steps are shown in Figure S20 and detailed in the methods). **A.** The number of cells in each semi-synthetic dataset (see panel D for legend). **B.** Percent of ϕ' that are significant plotted against the median UMI/cell of each semi-synthetic dataset. **C – D.** Purity scores of semi-synthetic datasets yielded using different feature selection methods. For each semi-synthetic dataset, either 2000 random features were selected or features were selected using HVGs, SCT, FvF or BigSur. Clusters were assigned using the Leiden algorithm (with resolution = 1) with 40 different random starting seeds and the purity was calculated. Points and bars denote median purities and interquartile range (IQRs) of purities, respectively. BigSur selected the top 10% of significant ϕ' for all semi-synthetic datasets except for those generated from the keratinocyte and 10k T cell datasets, for which the top 1% of significant ϕ' were selected. **E.** Empirical cumulative distribution function of the sequencing depths (total UMI/cell) of the acinar cells assigned to clusters 0, 1 and 4. **F – G.** For each of 20 semi-synthetic datasets generated from clusters 0 and 1 of the acinar dataset, the number of cells is shown in panel F, and the percent of ϕ' that are significant are plotted against the median UMI/cell are shown in panel G. For all except three datasets, in which two genes were found to have significant ϕ', no genes were found to have significant ϕ' (i.e. p-value < 0.05).

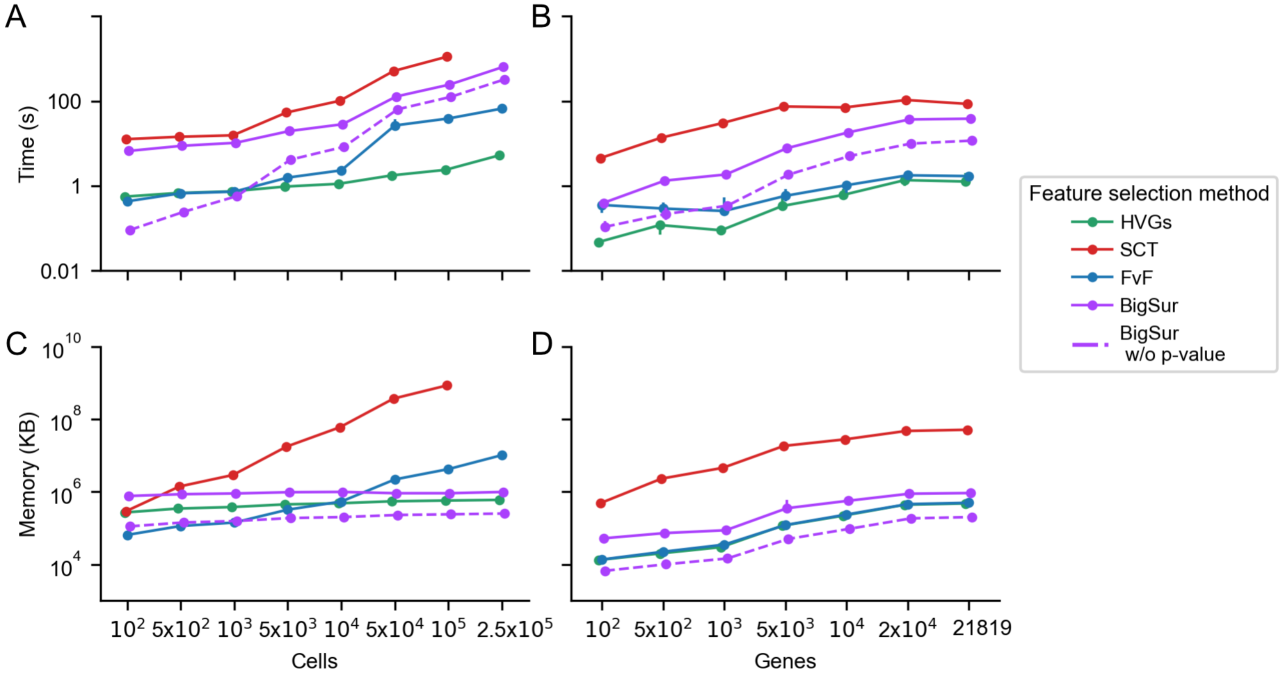

Supplementary Figure 21: Comparison of run time and memory usage of each method. The 1M PBMC dataset was downsampled to varying numbers of cells and genes and the total memory usage and run time of each feature selection was measured. For each plot, markers are medians and bars are interquartile ranges (IQRs).The time and memory usage of BigSur with (purple, solid line) and without (purple, dashed line) *p*-value calculation was measured. SCTransform was not run on datasets with 250,000 cells due to insufficient memory. **A – B.** Each method was timed for datasets with varying numbers of cells (panel A) and genes (panel B). **C – D.** The total memory usage of each method was measured for datasets with varying numbers of cells (panel C) and genes (panel D).
